# Analysis of factors affecting IoT-based smart hospital design

**DOI:** 10.1186/s13677-020-00215-5

**Published:** 2020-11-26

**Authors:** Banu Çalış Uslu, Ertuğ Okay, Erkan Dursun

**Affiliations:** 1 Engineering Faculty, Department of Industrial Engineering, Marmara University, Istanbul, 34722 Turkey; 2 BSH Turkey, Poligon Street, Buyaka 2 Residentials, 8, 1st, Istanbul, 34771 Turkey; 3 Faculty of Technology, Electrical and Electronics Engineering, Marmara University, Istanbul, 34722 Turkey

**Keywords:** IoT, Smart hospital, Smart hospital design, IoT layers

## Abstract

Currently, rapidly developing digital technological innovations affect and change the integrated information management processes of all sectors. The high efficiency of these innovations has inevitably pushed the health sector into a digital transformation process to optimize the technologies and methodologies used to optimize healthcare management systems. In this transformation, the Internet of Things (IoT) technology plays an important role, which enables many devices to connect and work together. IoT allows systems to work together using sensors, connection methods, internet protocols, databases, cloud computing, and analytic as infrastructure. In this respect, it is necessary to establish the necessary technical infrastructure and a suitable environment for the development of smart hospitals. This study points out the optimization factors, challenges, available technologies, and opportunities, as well as the system architecture that come about by employing IoT technology in smart hospital environments. In order to do that, the required technical infrastructure is divided into five layers and the system infrastructure, constraints, and methods needed in each layer are specified, which also includes the smart hospital’s dimensions and extent of intelligent computing and real-time big data analytic. As a result of the study, the deficiencies that may arise in each layer for the smart hospital design model and the factors that should be taken into account to eliminate them are explained. It is expected to provide a road map to managers, system developers, and researchers interested in optimization of the design of the smart hospital system.

## Introduction

A “smart hospital” is a concept that emerged as a result of rapid digitalization across the healthcare industries with the use of key enabling technologies, mainly Internet of Things (IoT), data analytics, availability of personalized services and Artificial Intelligence (AI). IoT is an ever-growing technology that has the ability to use distributed computing and the capability to exchange information to make rapid decisions for system needs within a vast distributed network. This technology connects everyday objects (smartphone, smart watch, smart light, etc.) such as sensors, actuators, and things to the Internet via existing networks to facilitate the diagnosis and follow-up of patients while increasing the efficient use of hospital resources. IoT applications are developed to use this connected network, relying on a digital environment. This offers new opportunities to provide fast and accurate responses by obtaining relevant information. This intelligent network can receive data from several sources, process data locally using the decreased computing power and/or in a centralized manner with higher digital computing resources to make smarter decisions. From this, intelligent recommendations, predictive analysis, or pattern detection can be made.

With these intelligent abilities, IoT technology also enables the improvement of Quality of Service (QoS). The information exchange is provided with a continuous flow between patients, doctors, pharmaceutical and biomedical suppliers, etc. In this sense, IoT uses advanced IT technology to integrate the various components of a collaborative network to improve the efficiency, service capability, and flexibility between smart devices. These smart devices can monitor and sense their environmental conditions, and measure the activities or the functions on the installed platforms. The gathered data then can be conveyed to a management unit/decision support system for further processing. Collected sensory data can be used to understand the system’s current situation by monitoring the states of each unit in the network and the status of the complete system. As a first step, data processing technologies can also be employed to transform raw into input data. Processed input data can be converted into meaningful information using information processing techniques and finally, this information can enable the system to provide self-action through knowledge processing approaches without human involvement [1]. In other words, IoT systems can create autonomous systems via self-governance and self-management abilities [2].

The IoT-based smart hospital studies are increasingly gaining interest in the literature. Numerous studies on IoT technology and smart hospital that propose new solutions and technological advancements have been published. However, there is no discussion has been found that handles smart hospital design as a holistic approach and explains it with a system development process that takes into account steps needed for each layer.

The main contributions of this paper can be summarized as;

- A brief history and overview of IoT technology are explained by demonstrating where IoT sits within smart hospital design.
- A five-layered IoT architecture is proposed, which can efficiently utilize optimization factors, challenges, available technologies, and opportunities, as well as the system architecture that comes about by employing IoT technology.
- Key technologies related to each layer are explained and compared and the impact on IoT architecture design.
- Possible future directions and research challenges are discussed.

**Fig. 1:**
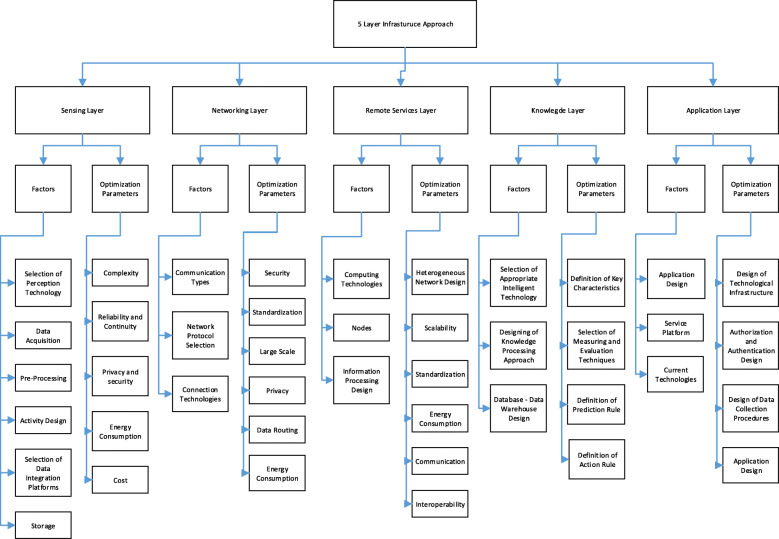
Taxonomy diagram of proposed five layered IoT architecture

Taxonomy diagram of proposed five layered IoT architecture is given in Fig. 1.

The rest of the paper is organized as follows: “smart hospital” and IoT-based applications is defined in “Literature review” section. Following that, “Smart hospital design” section handles the factors that need to be optimized for smart hospital design. In this Section, each layer is examined in detail, technological infrastructures are compared and their effectiveness is evaluated. “Conclusions and future perspectives” section gives some discussion points about IoT-based smart hospital design challenges.

## Literature review

The first definition of IoT was made by [3] at the beginning of the 21st century [3] and it officially appeared in literature for the first time in 2005 [4]. Health companies have begun using IoT technologies to make their business processes faster, more controllable, smarter, and more efficient. These technologies consist of three main layers: perception layer, network layer, and application layer [5, 6]. At that time, this novel technology (IoT) identified three main layers [7–12] considered as perception layer, network layer, and application layer. The perception layer was where the environment is sensed and the data is collected through the sensing devices and nodes. The network layer consisted of multiple wired and wireless network systems that provided the entire system with communication and interoperability among each other [6, 13]. The application layer enabled updating online servers based on the latest end-device values and provided integration of services and IoT applications [14, 15]. The basic capabilities of IoT was defined by Muralidharan [16] as;

1. Location Sensing,
2. Traffic Monitoring,
3. Environmental Monitoring,
4. Remote e-health Monitoring,
5. Remote Monitoring, and
6. Secure Communication and Ad-hoc Network

IoT is expected to contribute significantly to healthcare systems, and thus to have a positive impact on human health. Moreover, it was expected to improve processes that allow new diagnosis and treatment methods to be discovered and to accelerate patient access to patient related data. In addition to internal solutions, it was expected that all system stakeholders could have easier access to public health services and information on health systems. There are numerous researchers that have contributed to develop IoT-based smart hospitals in the literature. Some relevant examples are given below.

Park et al. [17] performed a study where IoT was used in different business environments (NFC and iBeacon which are Omni-channel services in hospitals) for enhancing health service quality. Diagnosing, planning, action and specifying learning phases were the four phases in their research. NFC and iBeacon designed 8 service models to find a solution for diagnosing problems in the hospital during the first two phases. In the third phase, they installed wearable beacons and NFC tags. In the fourth and fifth phases, the application process and the service models were evaluated by the stake-holders. These brand-new service models have significantly increased the efficiency of hospital staff and offered improvements in healthcare management.

The advantages/options provided by Wireless Sensor Network (WSN) technologies have spread over many areas of today’s modern life. RFID tags and sensors create systems using wireless networks. As we move from webpage to social networks, and the computing network, the need for data on demand has dramatically increased by using advanced heuristic queries. Increasing the need for data increases the number of data. In the model proposed by Park et al., big data will be analyzed via data mining and can be monitored via GIS-based imaging systems [18];

1. Architecture
2. Energy efficient sensing
3. Secure re-programmable networks and privacy
4. New protocols
5. Quality of service (QoS)
6. Participatory sensing
7. Data mining
8. GIS-based visualization
9. Cloud computing and international activities

Chaudhury et al. [19] proposed systems to monitor health-related parameters and communicate over wireless networks. In the event of an abnormality or an emergency, the system alerted appropriate staff and ensured the confidentiality and security of the patient through limited databases. Some of the sensors used in this system were temperature, pulse and motion sensors. The authors state that the telehealth system is efficient and user-friendly.

Rashed et al. [20] developed a medical platform for remote health monitoring systems. The concept of IoT proved remote monitoring with decreased residuals and decreased medical management expenses. Additionally, they found increased patient satisfaction and disease forecasting to improve treatment. Their IoT infrastructure was divided into three layers which were called perception, which included physical interface and data collection, network gateway and integrated application which included data analytic, data visualization, cloud and service- databases. Catarunucci et al. developed an IoT-based smart hospital system architecture for automatic monitoring of patients, staff and biomedical devices in hospitals [8].

With the increase of research in wearable technologies, a new technology called the Internet of Medical Things (IoMT) has emerged [21]. Some applications within this scope are patient monitoring [22], fall detection [23], detection for motion disorder [24], sleep monitoring [21], evaluation of illness degree of a clinical risk level [25], health monitoring [26, 27], medical image segmentation [28], attack detection [29], and implantable sensors [30]. Although this application required more expensive hardware but allowed for a more sophisticated service. In addition to applications on IoMT, another new paradigm implementation in order to examine people’s daily behavior and their interactions with their living environment is called as Healthcare Internet of Things (H-IoT). This technology includes machine learning (ML) and artificial intelligence (AI) integrated into the design and architecture of the entire system [31]. Some of the researches conducted in this area are blood pressure, heart-beat and glucose level [32–34], elderly healthcare [35], decentralized interoperable trust framework to enhances trustworthy factor (TF) estimation based on blockchain technology [36], estimation of under-five child mortality [37], and human activity recognition [38].

Hybrid sensing network provided information to an IoT smart gateway which included a two-way Proxy, a management application with a control database and a secure access manager with a user database. Then user interfaces provided environments for local and remote users via VPN servers [9]. Their study focused on the disadvantages of present hospital information systems and they proposed a scheme for a smart hospital based on IoT. The smart hospital was described with a third-grade hospital example that had the scheme of smart hospital and included its logic structure, framework application etc.. Ilin et al. [39] proposed a new digital business model in accordance with the services provided by health institutions. The business model defined smart hospital concepts.

Dhariwal and Mehta [40] proposed a smart hospital plan for using IoT at a built-in data point. The model proposed in the study highlights the importance of IoT for existing hospitals to be an effective healthcare provider. Providing a smart hospital brings a positive impact to the treatment mode in a doctor’s facility. Some other applications on smart healthcare are smart monitoring [41–45], IoT architecture for the health sector [46], healthcare frameworks [47–50], smart healthcare service management [51, 52], ubiquitous and quick access to personal health [53], equipment localization [14, 54], hospitalized patients and controlled drug consumption [55, 56], and false alarm detection architecture [57].

This section elaborates on current studies referring to smart hospital applications based on IoT technology. Although many studies address the partial optimization of factors and present the IoT system architecture, no holistic study has been found that evaluates design parameters and optimization factors together.

## Smart hospital design

**Fig. 2:**
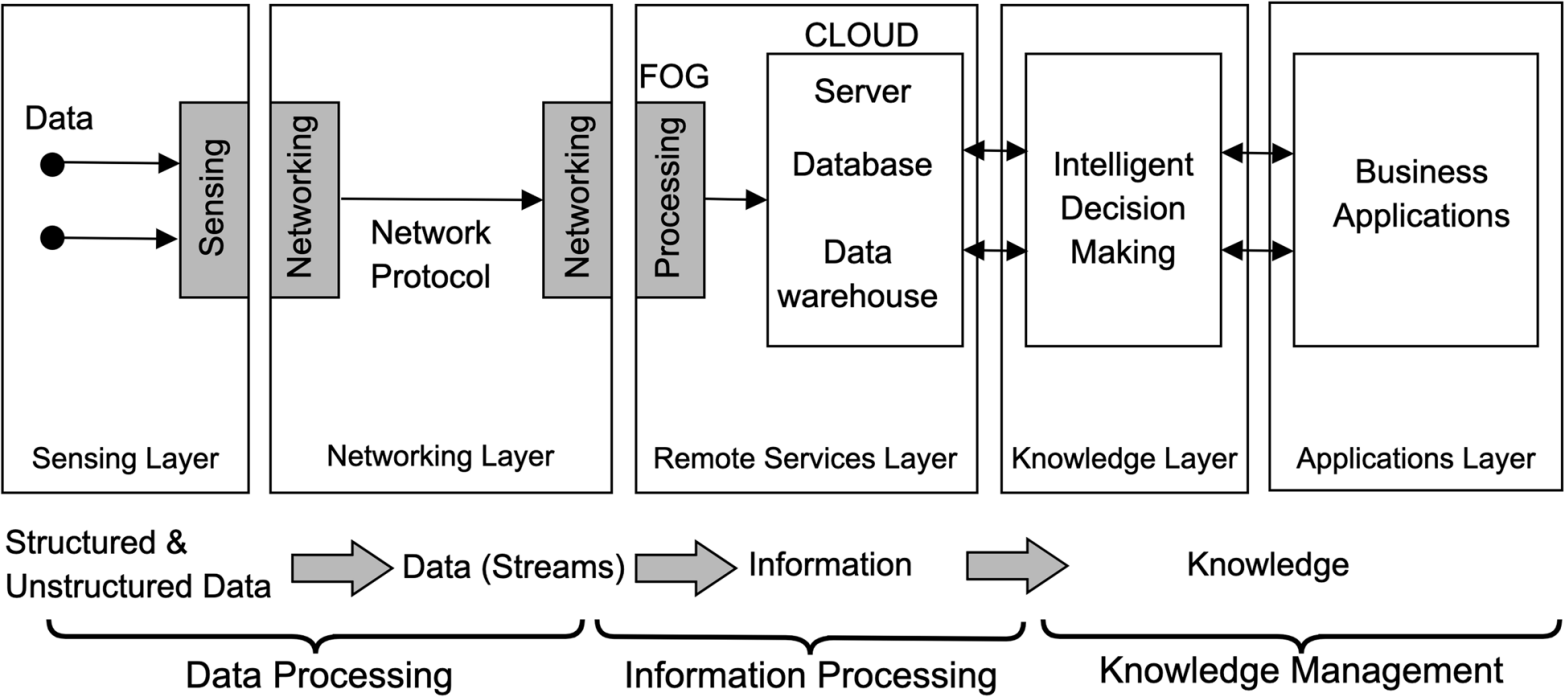
Transformation of raw data into meaningful knowledge based on IoT

In this study, analysis of the factors required to optimize IoT-based smart hospital design is analyzed based on five integrated IoT layers. The general approach in the literature is a 3-layer infrastructure approach. Three-layer structure is not sufficient for system design and modeling, and does not adequately reflect the system components. Although three-layer architecture is an essential structure for IoT technology [58, 59] and it enables to device to be connected to the internet [60], this architecture is not suitable for all applications due to high energy consumption, the low ability for integration and communication [61]. In addition to this, this architecture does not give a reliable solution [62] and hides many details related to functionality and data flow [63, 64]. In contrast, five-layer architecture offers lower capacities in storage and energy. Considering these functionalities, the five-layer architecture is more suitable for IoT applications. Due to the limitations and technologies required, the layers were separated here into five layers (see Fig. 2).

1st Layer - Sensing Layer: This layer includes data collection technologies that demonstrate the necessary system and application infrastructure as well as the acquisition of information at the points where the information is produced. Depending on the type, effectiveness and exchange of information needed by the stakeholders for the potential stakeholders of smart hospitals is included in this layer. It is aimed to provide an information exchange with different authorization and authentication levels defined for each stakeholder group. The structural or non-structural information that is produced, updated or which emerged for the first time is collected, made accessible and updated by the system.

2nd Layer - Networking Layer: This layer managing the transmitting data to the remote servers as well as to interconnect systems and platforms. 3rd Layer - Remote Servers Layer: represents the remote computational technology of the IoT system. 4th Layer - Knowledge Layer: this layer includes intelligent decision making and analysis module of IoT systems where knowledge processing is done. 5th Layer - Applications Layer: includes service platforms that are used by each systems’ stakeholders.

After each layer is identified, related factors that need to be considered in each layer are defined and illustrated in Fig. 2. From this point on, each layer structure is identified by the system approach and the optimization vision. While explaining the layer structures, the basic approach is to inform the readers about existing technologies. These steps that need to be completed in each layer and also about the limitations that will be faced while designing the layers. These limitations (constraints) define areas where a researcher can contribute to the planning and optimization of an IoT-based smart hospital design.

In this section, the proposed five layer smart hospital architecture is described in general. Each layer in the architecture is explained in detail in the following sections.

### Sensing layer

For a smart hospital design, the first step is to ensure that the stakeholders of the system are identified. Then, which data will be accessed by which stakeholder on the system and which data should be shared in the system should be analyzed respectively. Lastly, which data analytic methods will be used to analyze sensed data, including how the data will be collected from each stakeholder, should be defined. These steps are exactly a perception system design and do not only increase the quality of the service (QoS), but also ensure that the information required by each stakeholder is delivered in a timely and accurate manner.

The sensing layer specifies a way to monitor, store and analyze health data by employing ubiquitous and distributed computing technology. This layer points to nodes where data is generated and used at the primary level. At this layer, all of the stages of collecting data, determining the technology to obtain the data, the frequency of data collection and data analysis are all optimization problems. Another important factor is to determine how much of and when the collected and analyzed data should be shared with stakeholders. This problem is precisely the problem of system design. All system stakeholders need some of the information to be produced at the primary level in the system. For example, an insurance company would like to know the general health status of a person who wants to have life insurance. On the other hand, the hospital will want to quickly access the knowledge of how much of the diagnosis and treatment services will be offered to the person by the insurance company. It is important to make sure that these mutual information exchanges of smart hospitals are made in real time and that an adequate infrastructure is provided to ensure that relevant information is immediately transmitted to the system.

Sensing and recognition technologies can be divided into three types: wearable sensors, ambient sensors and location sensors. Some examples of these sensors are [57, 65, 66];

**Wearable sensors;** Electrocardiogram sensor - ECG; Resistor - LDR; GPS, Blood pressure cuff; Heartbeat Sensor: Sunrom-1157; Physiological sensors - Spirometer.

**Ambient sensors;** Temperature sensor (LM35); Light Dependent; Thermometer; Hygrometer; Noise detector; Humidity sensor; Motion detector

**Fig. 3:**
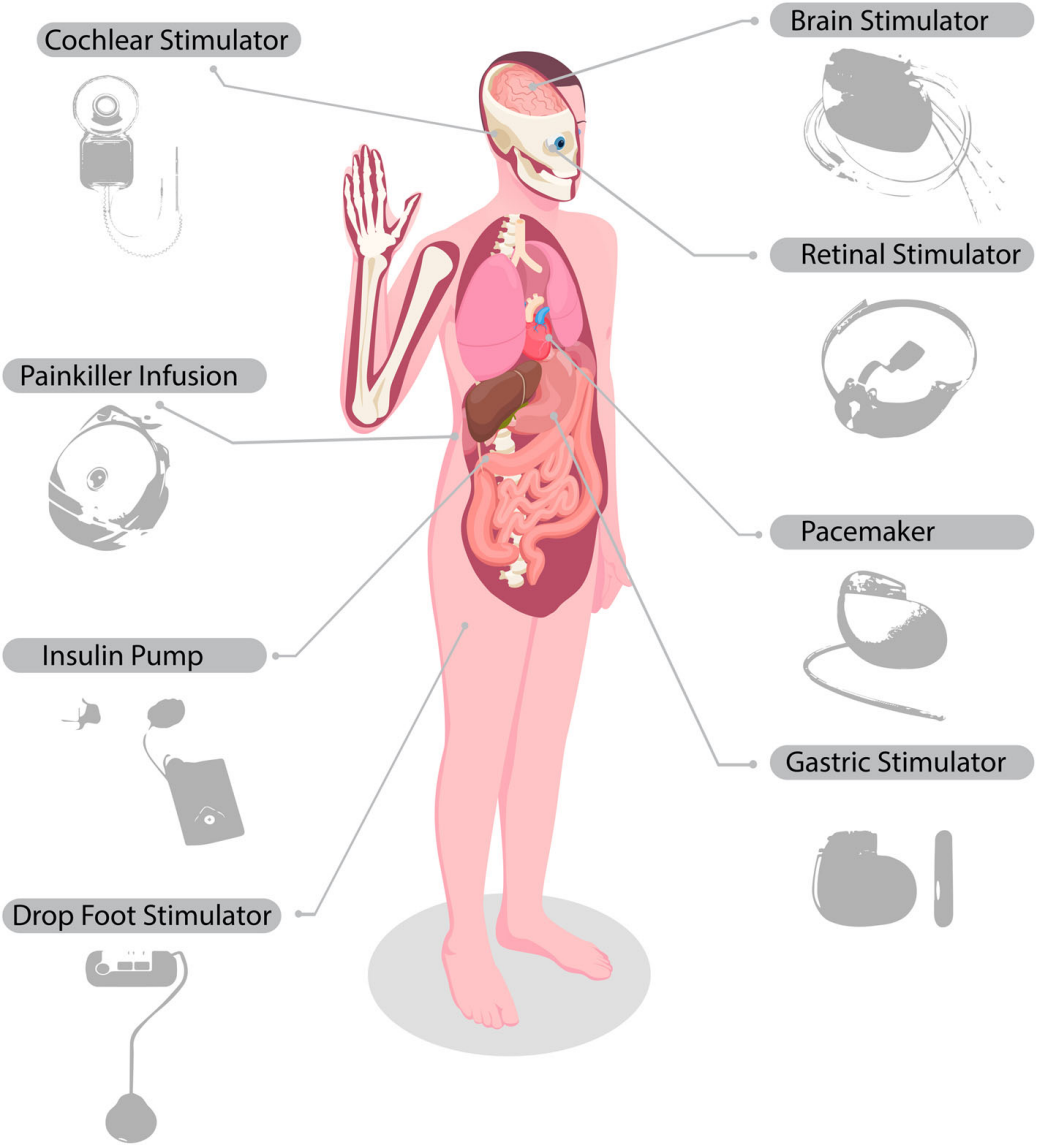
Some of implantable devices [67]

**Location sensors;** Infra-red, Zigbee, Active RFID; Binary sensors (Window contact, Door contact, Light switch, Remote control switch);

The other crucial components of the sensor layer are implantable devices as can be seen in Fig. 3. These devices are fully equipped with artificial intelligence algorithms without the need for doctor intervention, on the other hand, they can be used to transmit patient data to the smart hospital database.

**There are six main factors need to be considered in the sensing layer design;**

1. Selection of Perception Technology: In order to measure different factors such as motion, blood pressure and heartbeat, appropriate data collection technology must be selected (RFID, WBSN, HSN, MBSN, etc.)
2. Data Acquisition Design: It is required in numerous data collection procedures based on the three main criteria of data: 1) data structure (structured, Semi-structured or unstructured data), 2) on-time access (unreal -time, soft real-time, hard real-time) and 3) data sources (clinical, patient-generated, clinical research etc.).
3. Pre-Processing (optional): Preparing raw data for further steps via pre-processing stage (replacing missing values, cleaning, discretizing, etc.)
4. Activity Design: It is required to undertake a number of processes for planning, monitoring, diagnosis and evaluation in order to get Result-Based Management (RBM) system, (Qualitative methods, Quantitative methods, etc.)
5. Selection of Data integration platforms: To satisfy integration and data transmission into the network, it is required to select appropriate data integration platform. (Satellite, Mobile/static, Crowd sensed etc.)
6. Storage(optional)- It is necessary to build an appropriate storage design in order to record patients’ treatment reports and to share the data with other stakeholders such as doctors, insurance and IT companies.

Sensing layer factors that need to be optimized : These factors are design parameters and listed below:

1. Complexity: IoT consists of multiple devices working together in a heterogeneous network
2. Reliability and Continuity: It is necessary to ensure that these devices work together in a reliable and continuous manner.
3. Privacy and security: In this collaboration process, the privacy and security of the data must be provided.
4. Energy Consumption: The IoT network consists of smart devices that operate based on energy consumption and are designed to sense and react to changes in their environment.
5. Cost: While ensuring the interoperability of smart devices, it is also necessary to minimize the costs arising from requirements such as device, energy consumption, security.

The most critical issue in sensor technologies is the protection of personal data [68]. However, reliability of data, hacking of devices, device capacities, energy consumption, security design, cost, time and quality are also important factors for optimizing the efficiency of a smart hospital design [69].

The sensing layer of IoT consists of numerous sensing devices that can generate a large amount of real-time data. If this real-time raw data is uploaded directly to the application system, it will cause a decrease in the operating performance of the system, increase in complexity, delay in sharing perceived data, and increase in communication costs [70]. In order to optimize this complexity, edge computing can be used as edge-side services that processed timely and effectively near the sensing devices. The second important factor for the sensing layer is cost. In order to reduce the cost of data collection, the Raspberry embedded card system can be used due to its low cost [71], which allows taking photos of the patients in real time [72]. Another method is a service-concentrated information distribution method that can decrease in the time cost for service sharing but request processing time degrades the method’s performance [73].

Sensing layers with computation and decision-making capabilities in IoT systems are used in an integrated manner with physical world entities [74, 75]. Wearable technologies are started to use for assisting human movements [76]. The third and fourth important factors that need to be optimized are reliability and the continuity of the information obtained in this technology. Biophysical and biochemical signal collection technologies are used to monitoring health remotely, which can reduce time on the training of individual person and make the patient monitoring process more reliable than manual monitoring [77]. Wen et al. [78] emphasized that using energy harvesting approaches (TENG, PENG etc.) and storage (e.g., Super capacitor) approaches can optimize operations continuity.

The fifth important factor that needs to be optimize in sensor technologies is the protecting personal data [68]. Many different innovative solutions in this context have been proposed in the literature. Some of them are; [79] proposed a Blockchain-based solution that follows edge computing to guarantee privacy by considering regulation and users’ designed requirements. Sun et al. [80] proposed an optimized vector conversion approach and emphasized that by creating key generation, encryption and decryption stages with this approach, users can easily perform in the system without leaking any sensitive privacy. Schneble et al. [81] mentioned the necessity not only in technological but also in legal regulations, but they emphasized that the excessive severity of these regulations may negatively affect the researches.

The security of the data coming from different objects within the IoT system should be considered as the sixth factor to be optimized. In this context, to reduce the differences between data, [82] emphasized that it would be safe to use the k-anonymity method. Tahir et al. [83] proposed Integrated Circuit Metric (ICM) technology, but the critical issue of this method is generation time was not minimized. One of the most important issues to consider when trying to optimize the level of security is energy consumption. Because a highly level of security may lead to increase energy consumption and reduction in operating time [84]).

To optimize the efficiency of smart hospital design, considering all the above-mentioned optimization factors together is of great importance in increasing the design, quality and efficiency of the IoT system [69].

### Networking layer

The design of IoT networks specifies a way to transfer the packets from source to destination by using resource-constrained devices. The designed network is expected to operate for a long time to provide to collect, analyze and transmit huge data of the distributed system. For the definition of the network layer infrastructures, the analysis of the following four factors should be done. First, definition of communication types; second, selection of connection technology; third, construction of interoperability environment using networking technologies; fourth, selection of the true network protocol, fifth is defining the appropriate information processing approaches and the last one is optimization of the design parameters. Each factor is explained in detail below;

1. Communication Types: IoT devices (things or objects) can communicate with each other within the same or heterogeneous networks [85]. This communication between devices can be human to human (H2H), human to machine (H2M) or machine to machine (M2M) to analyze and produce information [86]. The main problem to enable a network that has communication ability is the lack of a widely accepted common platform that works with various applications so the network design should be standard to establish effective communication between subjects.
2. Network Protocol Selection: According to the information provided in the literature, well-known network protocols were analyzed and compared in accordance with the capabilities given in Table 1 [1, 87]. These protocols are;
  - CoAP: Constrained Application Protocol: Basically, it has 4 message types; 1. Confirm-able, 2. Non-confirm-able; 3. Acknowledgment; 4. Reset [1, 15, 88].

**Table 1:** Comparison of network protocols

Protocol	COAP	MQTT	XMPP	AMQP	DDS	HTTP	Web S.	SMQ
RESTful	yes	no	no	no	no	yes	yes	yes
Transport	UDP				UDP			
	TCP	TCP	TCP	TCP	TCP	TCP	TCP	TCP
Publish Subscribe	yes	yes	yes	yes	yes	no	yes	yes
Request Response	yes	no	yes	no	yes	yes	no	yes
QoS	yes	yes	yes	yes	yes	no	no	no
Security		SSL	SSL		DTLS			
	DTLS	TLS	TLS	SSL	SSL	SSL	WSS	HTTP
Header Size	4	2	NA	8	NA	NA	8	2
P2P	yes	no	yes	yes	no	NA	yes	no
Data Protocols	yes	yes	yes	yes	NA	yes	yes	no
Discovery	yes	no	no	no	yes	NA	yes	no
Scalability	yes	yes	yes	yes	yes	yes	yes	no
  - MQTT:Message Queue Telemetry Transport: Basically, it has 3 message types: 1. Fire and Forget; 2. Delivered at least once and 3. Delivered exactly before [89].
  - XMPP: Extensible Messaging and Presence Protocol: A platform supported by all Internet platforms [90].
  - RESTFUL Services: Representational State Transfer is a platform introduced by Roy Thomas Fielding in 2000 [91]. All features of http can be used on this.
  - AMQP: Advanced Message Queuing Protocol: There are basically three types of messages; 1. At most once, 2. At least once, and, 3. Exactly once [92].
  - Websockets: Websockets runs reliably over TCP and does not implement a reliability mechanism on its own platform [15].
  - HTTP: There are basically four types of HTTP message headers:1. General-header that can be used for both request and response messages. 2. Request-header that can only be used for request messages. 3. Response-header that can only be used for response messages. 4. Entity-header that defines meta information.
  - DDS: Digital Data Service: This protocol allows ad-hoc queries and filters in order to extract specific data [93]
  - SMQ: Simple Message Queues Protocol: Communication protocol of SMQ is Publish/Subscribe and it uses ad-hoc queries.
  The comparison of network protocols according to optimization parameters is given as follows [87, 94, 95]; It is the lowest power requiring COAP among the network protocols. However, COAP is weak in terms of security. It is very weak, especially in spoofing and amplification attacks. The highest power requirement occurs in the HTTP protocol. Because MQTT client must support TCP, this means it always keeps an connection open. MQTT is the ideal choice for multi-device networks. If resources are limited, COAP is the most appropriate option for point-to-point connections. The most crucial advantage of XMPP is its decentralized structure. XMPP works similarly to email and runs on a distributed network rather than relying on a single central server. This eases the security issue. Web sockets are the best option if we are building an application that needs constant real-time updates in smart hospital systems. Designed for sensors and actuators, SMQ is assigned a unique ID known as a temporary subject ID to each customer. This is an important feature for patient privacy. DDS consumes twice bandwidth than MQTT protocol. Although DDS uses twice the bandwidth of the MQTT protocol, it provides lower data latency.
3. Connection Technologies: Another important property of the network layer design is the selection of an appropriate connection technology that satisfies communication and interoperability between resource constrained devices. According to literature, these technologies are [96–106];
  - Personal area network (PAN): Well-known connectivity technologies on the personal area network are Bluetooth UWV and Zigbee. The basic application of personal area network is to monitor elderly patients in order to reduce intervention time, treatment and hospitalization costs.
  - Local area network (LAN): Well-known connectivity technologies on the local area network ais Wi-Fi. Also, basically LAN supports large numbers of sensors to transfer medical images, body motion sensing, posture assessment, and exchange of information between separate locations.
  - Wide area network (WAN): Well-known connectivity technologies on the wide area network are cellular (2G-3G-4G-5G) and low power wide area network. Basic applications of WAN are e-prescribing, alert systems, accessing patient records and information.

**Networking layer factors that need to be optimized:** These factors are design parameters and listed below:

1. Security: Digital systems are vulnerable to attacks;
2. Standardization: a large number of heterogeneous devices must be connected to analyze heterogeneous data;
3. Large scale: A large number of devices must be connected to other companies’ systems;
4. Privacy: Data confidentiality and authentication must be done for continuous information sharing.

The first optimization factor of network layer is network security caused by infested internet-connected things, and it has become a critical concern [107]. In order to optimize network security to minimize attacks, security vulnerabilities of the IoT system must be defined at both the device and network level. In this context, [108] divided IoT system security into three parts: device, field gateway, and cloud gateway. Later, the devices were classified (health, transportation, etc.) and defined the vulnerabilities with the type of attacks. In this direction, 18 different security control tools were identified (account timeout, two factor authentication, data encryption, etc.). The research also emphasized that limiting authorized persons and increasing privacy are important in minimizing the attack surface. Alshehri and Hussain [109] defined another essential element for IoT network security as enabling trusted communication among the nodes by a secure messaging system. In this context, the researchers proposed a model (IOT-KEEPER) capable of detecting and preventing network attacks and indicates that the model does not require sophisticated hardware or modifications and traffic analysis can be performing without significantly affecting network performance. Another critical issue is that the IoT system regularly expands by nature. As a result, new ones should be also expected to emerge regularly in addition to the existing security threats.

The second optimization factor is standardization. Standardization is a tool to guarantee the interoperability of different protocols from connected devices within IoT system [110, 111]. To ensure network standardization, protocols should be evaluated depending on the data formatting and message broker features in accordance with the IoT system.

The third optimization factor is optimal data delivery. The adaptive routing approach (ARA) can be used for optimal data delivery by considering routing paths across the IoT nodes. This method improves network efficiency by increasing resource utilization [112]. Another method of optimizing the data routing is done by [113]. It was emphasized that with the proposed method, it is possible to increase the transfer speed with minimum routing, and at the same time, it is possible to improve the throughput rate and provide security significantly. Yang et al. [114] also developed a real-time traffic network model based on the DBN (deep belief network) network model and the K-means and tested their model with real life application. The authors stated that the solution is optimal, closer to the practice, and fast convergence speed.

The fourth optimization factor is a large scale. In the IoT system, optimizing resource allocation is one of the key issues for large scale deployment. He et al. [115] proposed the alternating direction method of multipliers (ADMM) in order to solve resource allocation problems. The research includes a game-based resource allocation model that maximizes energy efficiency while minimizing network latency. Fang et al. [116] proposed an adaptive access control model that includes online machine learning and trust management features. The authors indicate that the proposed AI-based solution can reduce communication latency and increase control of security in the IoT system.

The last main optimization factor of the network layer is energy consumption. This factor aims minimizing the energy consumption of sensors in the IoT network in order to increase in the network lifetime. Iwendi et al. [117] selected the most appropriate Cluster Header (KH) in the IoT network to optimize energy consumption, considering many factors such as residual energy, cost function, and proposed a hybrid algorithm consisting of Simulated Annealing (SA) and Whale Optimization Algorithm (WOA). The authors state that based on the comparison results obtained from the proposed model and other optimization algorithms (Artificial Bee Colony algorithm, Genetic Algorithm, Adaptive Gravity Search algorithm, WOA), the proposed method yields superior results.

In addition to other optimization parameters, the high cost of wearable devices used in healthcare systems is one of the major issues especially, for remote monitoring of patients [118].

While establishing a robust connection technology, application developers who want to minimize their costs should make a comparison according to their system needs and choose the most appropriate connection and communication technologies.

### Remote services layer

The IoT remote services layer should handle the numerous simultaneous connected nodes (devices) which interact with each other in a distributed environment. In order to establish an efficient remote services layer infrastructure, three main factors must be considered by the design group. The first is computational technology, second is nodes which need to be placed to define increased interaction and third is design parameters. Each factor is explained in detail below;

1. Computing Technologies: Remote services based on models or methods facilitate distributed environments by ensuring digital computing for applications that require external computing and data warehousing. These applications are developed around the combination of some factors such as the amount of data which is gathered, analyzed and reported and simultaneous analytics. Cloud computing, fog computing, edge computing and mist computing are used to fulfill these requirements [119].
  Cloud computing is a combination of different fundamental features, three service types, and remarkable deployment models [120]. On-demand self-service, network access, resource pooling, rapid elasticity, measured service, utility computing, web services in the cloud and internet connection can be considered features of cloud computing. Service types are SaaS (Software as a Service), PaaS (Platform as a Service) and IaaS (Infrastructure as a Service). During smart applications, presenting data as accurate, reliable, energy efficient, and without latency, while protecting privacy and security are the basic building blocks of QoS [121–123], SaaS is a cloud computing model that enables users to use software, which is supported and developed completely by providers, without any money or time spending for installation, version upgrades and assistance of the software for users [124]. PaaS is a middleware cloud service model that allows developers to code, perform deployment and use testing platform in cloud infrastructure. It provides [125]. Developers can benefit from platform’s tools and interfaces to integrate the applications in providers’ environments with a high level of development and server/database resource allocation [126]. Iaas is a service delivery model that provides a large variety of hardware resources and on-demand services to manage remote services components (CPU, databases and server machines) via internet connection. This model ensures users improved capabilities and abilities in pay-per-use and on-demand provisioning [127]. There are four type deployment models, which are private cloud, community cloud, public cloud and hybrid cloud respectively [128]. This technology provides many specifications of numerous details for companies and users. However, these specifications can be a problem for applications which require nodes in surrounding environments in terms of latency and delay.
  In order to handle latency problems, the Fog computing platform which provides computing, data warehousing and connected services between end devices and basic cloud servers can be implemented to meet the requirements of analytic [129]. By applying and implementing fog computing, it is be possible to gain low latency, location awareness, improved quality of service and enhanced simultaneously synchronized applications due to the inclusion of end user devices, routers and switches [130]. In other words,Fog technology allows latency to be observably reduced when compared to cloud computing [131].
  The edge computing model enables able technological devices to perform computation at the edge of the network. Due to the fact that the amount of data produced at the edge increases gradually, processing and proceeding data at the edge of the network could be more efficient [132]. However, in system design and infrastructure of fog computing, edge devices are not involved in computation and data acquisition; therefore, they suffer from insufficient use of bandwidth and network delay [133].
  The last popular computational technology is Mist Computing. Mist computing pushes processing to the network edge to improve the autonomy of subsystems, reduces latency manages interactions of the complex system implementations. In mist computing, there are three elements called cloud, mist, which are managed by cloud server, and droplet, which is managed by mists [134].
  In terms of remote service technologies, when the system is designed in a distributed environment, end users and the devices are called sensing nodes. Sensing nodes work together by communicating with each other to provide interoperability in cluster nodes. These cluster nodes access a station where larger systems have been created, and as a result, much larger and decentralized systems can communicate with each other. With these distributed and clustered methods, the system can be designed more easily and more effectively. At the design stage of the remote services layer, developers will need to work on the Heterogeneous Network Design. The designer who wants to optimize the interactions is expected to consider the constraints of scalability, standardization, energy saving, communication and interoperability constraints in order to design effective and efficient computational technology [69, 131, 135].
2. Nodes: In order to design the remote services layer, a second step is to identify central, cluster nodes and a base station. Optimization factors that need to be considered, while doing this analysis are; [136].
  Reduce energy consumption,
  Satisfy communication of data over shortest distance,
  Provide respective cluster head, and
  Eliminate as much network contention as possible
3. Information Processing Design: In order to satisfy reliable information processing, the designer should consider the following; 1. accuracy, 2. trustworthiness, and 3. provenance of IoT streams [130]. Information processing, defines the robust and accurate information capturing. The security of the image and information capture system is very important in terms of detecting and recording accidents, losses and thefts in visual data [137]. GIS-based (geographic information system based) visualization includes data acquisition, data storage and data manipulation from multiple resources and it is accepted as one of the key component in decision making system in order to offer accurate and reliable data related in healthcare management [138–141].
  Tuli et al. [142] proposed a model that can achieve low latency and high accuracy with the integration of deep learning and IoT technologies in edge computing in health management. They presented a structure called Fogbus in the model, developed to increase the operation time and accuracy. The authors emphasized that the prediction accuracy of the model they proposed was 80% confidence. Patan et al. [143] proposed a Deep Neural Network-driven IoT smart health care method for real-time data analytics. The authors emphasized that with this method, it is possible to perform an efficient and accurate analysis of medical data, and that they achieved an accuracy of 88.88% with the proposed model. Cheng et al. [144] indicate that SVM gives the best classification performance in terms of accuracy and also allows much place to classification of future data.
  If the accuracy and trustworthiness of the medical data obtained is not be guaranteed, using this data for treatment purposes can cause major problems for healthcare system [145]. In this context, ensuring the data provenance enables the data flow on the system to be controlled and recorded and monitored [146]. Elkhodr and Alsinglawi [147] have developed a provenance-based trust management solution that enables to measure the trustworthiness of information obtained from a specific smart device numerically. Authors were identified a four-stage trust score value in data Provenance module; Trust value 0 indicates that device cannot be verified, Trust value 1 shows that device passed the verification process. Trust value 2 and 3 were calculated based on the result of data mining and AI algorithms in proposed model.
  Ensuring the accuracy and reliability of the data streams from the patient is even more vital in health services such as intensive care and emergency situations. Olokodana et al. [148] have proposed a real-time automatic seizure detection system. The proposed model has developed an epileptic seizure detector using the Ordinary Kriging Method based on the Edge computing method. The authors emphasized that the training accuracy of the proposed model was 99.4%.
  Due to the involvement of human life, a sufficient level of accuracy and high reliability for this information is needed. So, numerous processes to examine data sets in order to draw true information are required. These processes include not only data collection and analysis but also integration of data through the creation of a common format in a network system (Big Data Analytics Data Streams, etc.).

**Remote Services layer factors that need to be optimized:** These factors are design parameters and basically, six design parameters should be considered during the design phase of this layer. These are, respectively,

1. Heterogeneous network design,
2. Scalability,
3. Standardization,
4. Energy saving,
5. Communication, and
6. Interoperability.

The optimization of these 6 constraints is essential to ensure the design of a structure that can enable the full access of IoT devices in different locations to the server.

Communication between heterogeneous networks in the IoT system must be optimized through an integrated exchange of information. Yang et al. [149] proposed a joint optimization model to minimize the total missing probability. Their research, presented a model that optimizes the content placement and activation densities of base stations (BS) at different layers under the base cache size and BSs energy consumption cost constraints. The simulation results, emphasized that the co-optimization of content placement and BS activation densities is superior to content placement optimization alone. Shafique et al. [150] emphasized that it is possible to implement the IoT vision with 5G technology to integrate access in heterogeneous networks. The authors emphasized that with 5G technology, BS allows a vertical beam to each mobile device and it is possible to provide multiple access to the devices through the beam division multiple access (BDMA) technique.

The second optimization factor is the scalability that defines the ability of a device to adapt to the changes and ability to change based on IoT system’s future needs. Zyane et al. [151] suggested combining the traffic-oriented mechanism and the resource-oriented mechanism with adaptation actions using autonomous middleware for scalability management. This method, aimed to maximize the number of requests received by providing an acceptable QoS level and system performance. They emphasized that the proposed approach gives better results when compared with the methods than those mentioned as a reference. Aftab et al. [152] examined the scalability analysis of a LoRa network using stochastic geometry. When the authors examined the results obtained from the spectrogram graph, they emphasized that when there is more than one node with the same spread factor, the transaction cannot be correctly resolved by the receiver.

The third optimization factor is the standardization of protocols and approaches coming from connected devices. Standardization aims to ensure that the IoT system’s device work together at a manageable and scalable level. It may be possible to reduce the gap between protocols and reduce both transportation and data costs [153]. There are many standards developed within this scope. Rafique et al. [154] analyzed these standards in detail by gapping them according to their characteristics. The authors emphasized that for the protocols in the IoT system, resource constraints should be considered first and standardization are of vital importance to ensure interoperability.

Transforming an energy-efficient service into a time-efficient system is a fundamental issue of remote service design. Zang et al. [155] proposed an algorithm based on constrained Markov decision processes to optimize the energy efficiency for remote health monitoring. The authors indicate that the proposed algorithm leads to 100% throughput improvement in various power consumption budgets. Yang et al. [156] emphasized that using the Markov state transition theory, random transmission path selection can reduce energy consumption while at the same time ensuring safe data transmission. Emam et al. [157] emphasized that the transmission of medical data can be optimized by reducing energy consumption and system delay in IoT systems developed using edge computing, while [158] states that increasing the size of each fog will cause an increase in cost and energy consumption accordingly. Therefore, the authors emphasized that the fog size that should be used should be optimized depending on the developed model.

Inadequate connection with existing network technology is one of the key issues affecting communication and interoperability. Therefore, communication and interoperability in remote health systems are directly integrated with heterogeneous network design and standardization of the IoT system and need to be addressed together. Many efforts were addressed addressing the communication and interoperability problems caused by the information silos arising from the diversity of the IoT system in the field of Electronic Health Records. Aburukba et al. [159] emphasized that fog computing having a small communication delay, and when compared to the latency deployment feature of cloud and fog computing technologies, cloud computing has high communication latency deployment. Jaleel et al. [160] proposed a novel framework that integrates cloud, edge, and fog computing technologies in order to provide interoperability and optimal bounded requirements. The authors stated that this horizontally integrated medical IoT system also provides optimum resource utilization. Also, another critical issue is to minimize communication errors. In this context, [161] stated that it would be possible to minimize communication errors using the incorporated recurrent learning process.

In this section, detailed information about the remote services layer is provided. In this context, it is necessary to decide on the most appropriate technology in line with the system needs to ensure the effective management of a large number of simultaneously connected nodes (devices).

### Knowledge layer

Smart health systems have undergone many changes in areas such as manual processing, mechanization, automation, information, integration and intelligence. Especially, advances in the field of robotics and Artificial Intelligence (AI) have emerged as key technologies in patient care and disease management [162].

Specifically, AI applications have made significant progress in personalized risk assessment, disease diagnosis and image processing for patients [163, 164]. In addition, integration of AI technologies and big data analytic methods that include machine learning, game theory, optimization algorithms, among others, have enabled the creation of autonomous IoT structures that can self-repair, self-heal, capable of self-protect and self-organize. These capabilities have been used to make timely decisions to system needs as the way of working is getting simpler and easier [165]. In this layer, 4 main factors need to be considered, these are;

1. Selection of appropriate intelligent technology: In order to design appropriate intelligent decision-making systems, the designer should answer of the following questions;
  - When is the intelligent decision required?
  - Which type of intelligence is required for a smart hospital and why?
  - Which type of devices are needed?
  - Who needs to see intelligent results and who needs to be intelligent?
2. Designing of Knowledge Processing Approach; This step includes selecting, composing, and integrating computational technologies in order to take automated actions and identify predictions based on the results of processed data by using cognition ability. Some of the methodologies are listed below;
  - Data-Driven Approach (i. Supervised learning methods, ii. Semi supervised learning methods, and iii. Unsupervised learning methods
  - Knowledge based approaches
  - Hybrid approaches
3. Database - Data Warehouse Design: In this step, the designer should create an efficient data management model that is composed of centralized or distributed database or data warehouse to design online analytical queries and processing databases. The main issue for an appropriate data warehouse design is to translate all types of healthcare data into a common format to integrate with other warehouses. Other issues are: being cost-effective and an ability for ad-hoc exploration for large data sets.
4. Database - Data Warehouse Design: In this step, the designer should create an efficient data management model that is composed of centralized or distributed database or data warehouse to design online analytical queries and processing databases. The main issue for an appropriate data warehouse design is to translate all types of healthcare data into a common format to integrate with other warehouses. Other issues are: being cost-effective and an ability for ad-hoc exploration for large data sets.

**Knowledge layer factors that need to be optimized:** These factors are design parameters and basically, four design parameters should be considered during the design phase of this layer. These are, respectively,

1. Definition of key characteristics,
2. Selection of measuring and evaluation techniques
3. Definition of prediction rule
4. Definition of action rule

Knowledge layer design is essential for managing the interaction between devices in a distributed network [166], providing computational intelligence [167], and enhancing the intelligent decision-making ability of the IoT system [168]. Data acquisition from different devices in the IoT system is possible in two ways, structured or unstructured. Rathee et al. [169] states that well defined intelligent decision making system can lead to increase system ability on more structured, secure and efficient data acquisition mechanism.

In this decision making system, ensuring the traceability of all information is another critical issue in order to optimize system performance [170]. Urbano et al. [137] defines the traceability with 3 parameters consisting of capturing, storing and transmitting information. These parameters are the key characteristics of the learning module of decision making system responsible for many situations/solutions. For this reason, they should be designed to be the most suitable for the structure of the system. Another characteristic that needs to be optimized is the machine understanding. Guo et al. [171] proposed that semantic technology that includes semantic annotation, reasoning and service based on associations for machine understanding in order to fulfill interoperability in IoT system. The third important optimization characteristic is a learning method in the decision making system. There are different types of algorithms in order to perform learning. Some of them are decision tree, logistic regression, association rules, deep learning, clustering algorithms, and support vector machines (SVM) [171–174]. Researches and academicians mostly use all these learning algorithms but in real life applications, it is not possible to propose one algorithm due to diversity of IoT applications. The fourth characteristic is knowledge representation. Sanin et al. [175] defined the characteristics required to knowledge representation as follows; standardization, versatility and being dynamic, storage with appropriate configuration, transportability and share ability and predicting capability. All details in these three factors should be designed in the most appropriate way for the structure of the IoT system.

### Application layer

This layer is composed of practical applications that are designed for each stakeholder in order to deal with the amount of data and enable services for a smart hospital. The design of this layer includes three main steps that are:

1. Application Design: Data privacy is an elemental factor that should be guaranteed in the health sector and in smart hospital systems. Collected and gathered data are combined in a central database but all of them must be inaccessible to users. In order to achieve this, the application design is proceeded by analyzing authorization and authentication factors in detail while designing applications.
2. Service Platform: This platform defines different practical applications at the level of different authorization and authentication. For a smart hospital, nine different applications must be designed. These are:
  - Doctors and Physicians
  - Emergency Care Unit
  - Insurance Care Unit
  - Patients
  - Management
  - IT Companies
  - Pharmaceutical Companies
  - Biomedical Companies
  - State Institutions
3. Current Technologies: Current healthcare application technologies are listed below:
  - M-health: M-health is an e-health field where mobile devices and wireless devices (WiFi), global positioning system (GPS) and wireless technologies such as Bluetooth are used to support medical and public health practices.
  - E-Health: This technology focuses on using IT-based information and communication technologies in the healthcare and medicine industries [176].
  - Telemedicine: Telemedicine is a technology used by all health professionals to use information and communication technologies to exchange information for diagnosis, treatment and prevention [177].
  - P4- Medicine: The P4-Medicine approach is currently increasing, which is termed predictive, preventive, personalized, and participatory [178, 179]. This approach is operated by systems strategies and new technological developments for disease diagnostics, therapeutics and prevention, coupled for digitalization of the health sector [180]. Mobile Medical Doctors Assistants: Mobile Medical Doctors Assistants is a project that allows interaction between medical professionals and biomedical data [181, 182].

**Application layer factors that need to be optimized:** These factors are design parameters, and basically, four design parameters should be considered during the design phase of this layer. These are, respectively,

1. Application design
2. Design of data collection procedures
3. Authorization and Authentication design
4. Design of technological infrastructure

The application layer offers graphical interfaces based on the information received from the lower layer in order to perform all services including sensing and actuation by interacting with end-users [64]. Through this layer, all users can access reports, data related graphs, and data flow graphs. These applications must adapt to environmental changes [183] and dynamically receive and record the information in patients’ health status [184]. Through this layer, too many users access the healthcare IoT system, with many distributed different devices simultaneously. Therefore, Authentication, authorization, and data delivery procedures need to be identified and optimize based on the user to ensure that the right user can access the correct information [185]. In addition, it is recommended to use lightweight cryptographic algorithms in order to define confirmation and key understanding in this layer [186].

### Discussions

This research deeply analyzes the design of the smart hospital. In so doing, an important contribution has been made by adopted a holistic model of the structure, focused upon the smart hospital, a well-defined optimization factors and interpretive flexibility, and under-researched research context. Moreover, by explicitly recognizing that optimization factors have a significant impact upon a design, we have attempted to provide a complete account of the smart hospital design structure powered by IoT capabilities.In order to that, a five layered architecture of IoT-based smart hospital designed is analyzed, providing a detailed analysis on the design steps, challenges, technologies and methodologies for effective process design. The layered architecture is comprised of a 1) sensing, 2) networking, 3) remote services, 4) knowledge and 5) application layers, which altogether provide end-to-end connectivity and a complete solution for information exchange within the system. Moreover, a specifically smart hospital was analyzed and studied as a use case. IoT technology and its effects on the smart hospital were also analyzed. In this work, we optimized functions, designed processes and developed key technologies which were also compared.

**Fig. 4:**
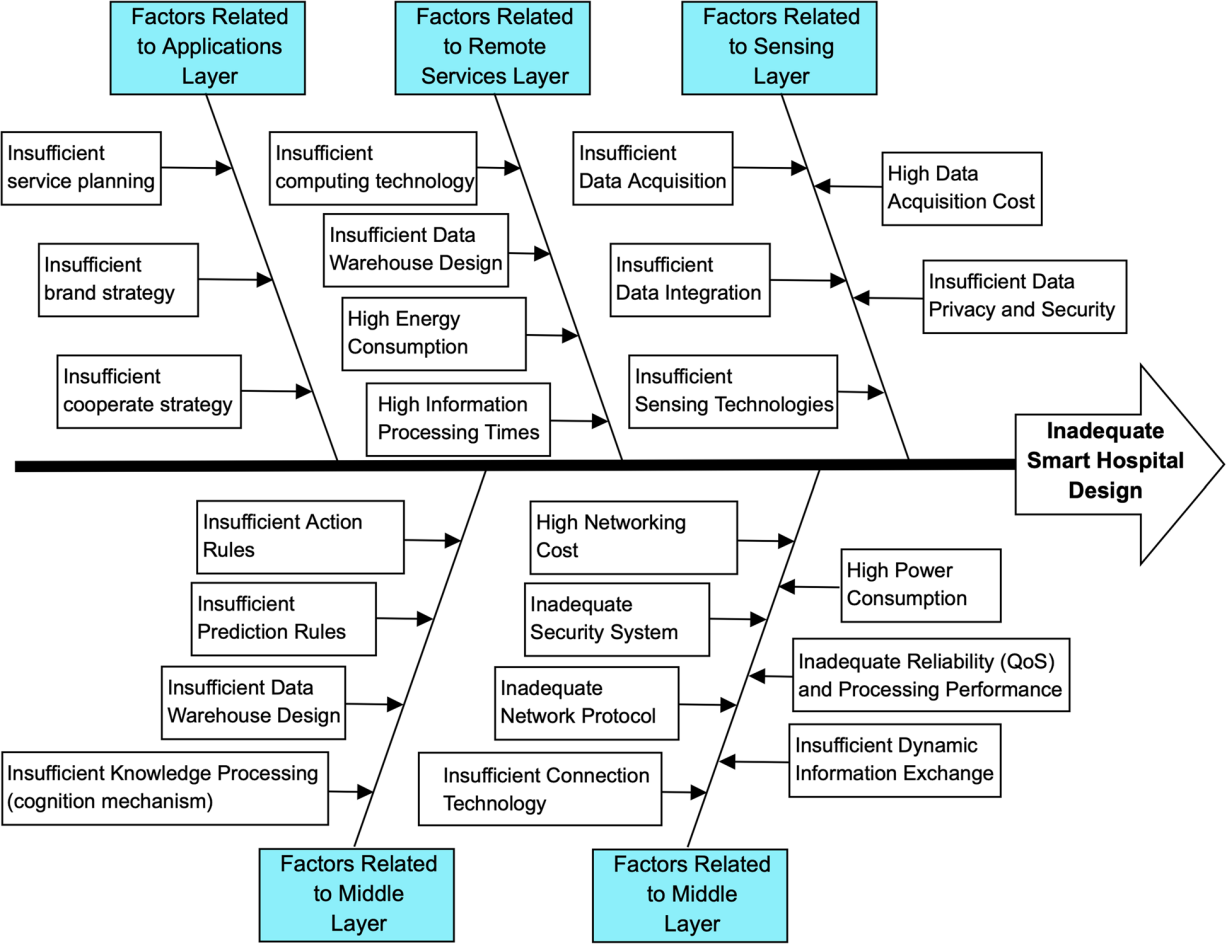
Cause and effect diagram for inadequate smart hospital design

For an IoT-based smart hospital design, the inadequacies that may arise in each layer and the resulting shortcomings in design are summarized in Fig. 4. To avoid inadequate smart hospital design, data from the IoT network must be collected continuously and safely. Data acquisition and integration methodologies must be identified appropriately for the big data environment in IoT applications as well. Then data acquisition cost, data privacy and security must be controlled. Data need to be recorded periodically and need to be transferred using networking technologies. To achieve this, data need to be adapted suitably in the sensing layer. In the networking layer, appropriate network protocols and connection technologies must be selected considering reliability, cost, power consumption and security constraints. In the remote services layer, related semantics to define the data models must be identified and integration of heterogeneous data sharing must be supported. Here, information processing must also be satisfied via distributional processing, database management and data appraisal. After the information is gathered, knowledge processing in the IoT system must be designed as a knowledge layer to produce knowledge via intelligent decision-making mechanisms. At this stage, selecting appropriate knowledge processing algorithms is essential to design efficient cognition mechanisms. Finally, application platforms must be adequately designed to share produced knowledge at the authorized level.

At the stage of converting the data obtained within the network infrastructure into meaningful information, hospitals need to manage a large amount of data and data mining is becoming an appropriate way to analyze it. Data mining techniques used in business is as follows;

1. Classification is used to determine categorical class labels using training data sets and predefined characteristics of data [187, 188]. Some methods that can be used for classification are;
  Neural networks
  Decision trees
  Logistic regression
2. Clustering is used to create “meaningful natural” clusters that contain similar data points but dissimilar data points with other clusters, and it is an unsupervised learning technique used in customer segmentation widely [189, 190]. Some methods that can be used for clustering are;
  K-means
  Kohonen network
  Divisive hierarchical clustering
3. Association rules is used to define effective association rules for huge data sets [189, 191]. Application steps of association rules are;
  Defining of the large item sets
  Usage of the sets in order to get association rules

Afterwards, necessary network technologies are analyzed to establish the necessary infrastructure below. This infrastructure is used to collect data and create a smart system that can work together within the network.

Detail comparison of connection specifications of some of the network types are; [96–106, 192–195];

1. The maximum signal rate that can be reach using PAN, LAN and WAN networks are;
  PAN: 250 kb/s-2 Mb/s (Bluetooth, Zigbee),
  LAN: 433Mb/s (Wifi),
  WAN: 50kb/s, very high level depends on generation (Cellular-3G,4G, LPWAN (Low power wide area network)
  in order to satisfy maximum signal rate it is necessary to use LPWAN.
2. Nominal TX power that can be reach using PAN, LAN and WAN networks are;
  PAN: 0-10 dBm, -25 to 0 dBm (Bluetooth, Zigbee),
  LAN: 15-20 dBm (Wifi)
  WAN: 20 dBm, depends on generation (Cellular-3G,4G, LPWAN (Low power wide area network)
  in order to get maximum output power (in dBm) that fed to the antenna,either WAN or LPWAN should be selected.
3. Power consumption for PAN, LAN and WAN networks are;
  PAN: Medium, very Low (Bluetooth, Zigbee),
  LAN: 15-20 dBm (Wifi)
  WAN: High, very low (Cellular-3G,4G, LPWAN (Low power wide area network)
  in order to reach low power consumption, either Zigbee or LPWAN should be selected.
4. Battery life for PAN, LAN and WAN networks are;
  PAN: Several days (Bluetooth, Zigbee),
  LAN: Several hours (Wifi)
  WAN: Low, long (Cellular-3G,4G, LPWAN (Low power wide area network)
  in order to reach high battery life, LPWAN should be selected.

Detail comparison based on the communication specifications of some of the network types are; [96–106, 192–195];

1. Nominal range that can be reach using PAN, LAN and WAN networks are;
  PAN: 10m, 10-100m (Bluetooth, Zigbee)
  LAN: 10-100m (Wifi)
  WAN: 2km-35km, 2km-20km (Cellular-3G,4G, LPWAN (Low power wide area network)
  In order to reach high nominal range, LPWAN should be selected.
2. Max number of cell nodes that can be reach using PAN, LAN and WAN networks are;
  PAN: 8, greater than 65000 (Bluetooth, Zigbee)
  LAN: 2007 (Wifi)
  WAN: Very high, very high (Cellular-3G,4G, LPWAN (Low power wide area network)
  In order to reach max number of cell nodes, either Zigbee or WAN networks should be selected.
3. Real time propriety of PAN, LAN and WAN networks are;
  PAN: Yes, yes (Bluetooth, Zigbee)
  LAN: Yes (Wifi)
  WAN: Yes, yes (Cellular-3G,4G, LPWAN (Low power wide area network)
  There is no significant difference between network types.
4. Success Metrics of PAN, LAN and WAN networks are;
  PAN: Cost convenience, Reliability and Power (Bluetooth, Zigbee)
  LAN: Speed and flexibility (Wifi)
  WAN: High data rate, coverage, reliability, noise-free (Cellular-3G,4G), Coverage, low power consumption, cost, scalability (LPWAN (Low power wide area network)
  Based on the success metrics, LPWAN should be selected.

Detail comparison based on the interoperability specifications of some of the network types are; [96–106, 192–195];

1. Number of RF channels for PAN, LAN and WAN networks are;
  PAN: 79, R1/10;16 (Bluetooth, Zigbee)
  LAN: 14 (Wifi)
  WAN: greater than 100 depends on generation, Depends on implementations (Cellular-3G,4G, LPWAN (Low power wide area network)
  Based on maximum number of RF channels, WAN should be selected.
2. Channel bandwidth for PAN, LAN and WAN networks are;
  PAN: 1MHz (Bluetooth), 0.3/0.6 MHz to 2 MHz (Zigbee)
  LAN: 20MHz, greater than 20 MHz by channel aggregation (Wifi)
  WAN: Depends on generation, 125 – 500 kHz (Cellular-3G,4G,LPWAN (Low power wide area network)
  Based on channel bandwidth, WAN should be selected.
3. Data protection and error control for PAN, LAN and WAN networks are;
  PAN: CRC (16-bit) (Bluetooth, Zigbee)
  LAN: CRC (32-bit) (Wifi)
  WAN: Four different channel coding; CS1, CS2, CS3 and CS4 that Max. throughput from 9.05 to 21.4 and payload from 181 to 428 (Cellular-3G,4G), AES: minimum key size is 128-bit (LPWAN (Low power wide area network)
  In terms of data protection and error control, it should be selected based on implementation and size

Lastly, In order to construct an intelligent decision making and analysis module, it is necessary to use artificial intelligence techniques in IoT applications [196]. These applications give an ability of cognition to smart devices in order to create intelligent decision-making mechanism. in this respect, Some of the methodologies are listed below;

1. Data-Driven Approach
  Supervised learning methods; Zhu et al. stated that calibrated boosted trees give the best result as a learning algorithm overall [197]. Zhou et al. do another comparison analysis; their analysis represents that random forest (RF), and gradient-boosting machine yield the better result [198].
  Semi-supervised learning methods: This method is a kind of machine learning algorithm. the main issue related with this approach is scalability [199]. Some methods are; generative mixture models, co-training, self-training, and transductive support vector machine [200].
  Unsupervised learning methods. Well-known unsupervised learning algorithms are 1) Clustering, 2) Anomaly detection, and 3) Neural Networks.
2. Knowledge based approaches: These methods include defining of perception, decision and action mechanism in order to create a business outcome. There are numerous studies on knowledge-based approach in the literature [201–205].

In this section, analyzes based on additional information are presented in line with selective high-quality research articles published in the field of IoT-based smart hospital design. It is expected that the findings of the research will be a guide for designers, researchers, and decision makers.

## Conclusions and future perspectives

The article has presented a holistic analysis of factors to smart hospital design based on IoT technology. This technology provides a bridge between business applications and computerized nodes in the heterogeneous network. IoT technology provides great opportunities for smart healthcare systems in order to develop and manage wearable technologies, real-time information sharing, patient monitoring, and vital readings applications.

Successful usage of AI techniques in order to interpret and analyze collected data is inevitable. The most challenging problems to design a smart hospital are to create a standard protocol design, to implement robust security and privacy design, to manage heterogeneous devices and to use resources efficiently. In order to create an effective IoT design, an optimization of the power consumption needs to be highly considered. From the analysis and design, we can conclude that our architecture will be very helpful to create an interoperable smart hospital design step by step, but further research and experimentation is needed.

As seen in the outbreak of COVID-19, in case of epidemic diseases that affect life on a global scale, the disruptions and human-oriented errors that will occur in hospital management systems make it difficult to control the epidemic. Therefore, IoT-based hospital systems can prevent the spread of the epidemic on local and global scale by reducing human-to-human contact.

Future efforts focus on the standardization of communication between IoT devices. This still poses a major challenge for IoT technology, and so the continuation of research in this area will be meaningful.

The improvable aspects of the proposed study for the design of IoT-based smart hospitals should be evaluated under two headings. The first is the situations where the patient has to come to the hospital, the second is the situations when the patient does not come to the hospital. The patient’s registration to the appointment system may be shaped by several questions such as how the patient will come to the hospital, which health unit the patient will come to, and what tests will be performed after the examination. Under this heading, smart parking systems can be developed for a smart hospital. The patient registration system can reserve a parking space for the patient. In this system, the numbered parking lot in the parking is reserved by using a mobile application before the patient reaches the hospital. We can control this through a remote controlled lock system. Second, we can develop different scenarios for situations where the patient will not come to the hospital, such as remote monitoring of the physiological and biological changes of patients. IoT structures that allow patients to be monitored remotely should not allow errors or should work with minimal errors. Such structures, which will occur in smart hospitals, should be evaluated with many parameters such as low latency in data transmission and high real time processes. The expected features of IoT systems and infrastructures in smart hospitals are high battery lifetime, high reliability, high bandwidth, ultra-low latency, interoperability (standardization), EMI-EMC ability, security-trust-privacy (STP), low-cost and low-power communication.

## Data Availability

Not applicable.

## References

[1] Al-Fuqaha A, Guizani M, Mohammadi M, Aledhari M, Ayyash M (2015) Internet of things: A survey on enabling technologies, protocols, and applications. IEEE Commun Surv Tutorials 17(4):2347–2376.

[2] Sterritt R, Hinchey M (2005) From here to autonomicity: Self-managing agents and the biological metaphors that inspire them In: Integrated Design & Process Technology Symposium (IDPT 2005), 143–150.. Society for Design and Process Science, Beijing.

[3] Ashton K, *et al* (2009) That ‘internet of things’ thing. RFID J 22(7):97–114.

[4] Strategy I, Unit P (2005) ITU Internet Reports 2005: The internet of things. Geneva Int Telecommun Union (ITU) 1:62.

[5] Santoro G, Vrontis D, Thrassou A, Dezi L (2018) The Internet of Things: Building a knowledge management system for open innovation and knowledge management capacity. Technol Forecast Soc Chang 136:347–354.

[6] Uslu BÇ, Fırat SÜO (2019) A Comprehensive Study on Internet of Things Based on Key Artificial Intelligence Technologies and Industry 4.0. In: Bouarara HA, Hamou RM, Rahmani A (eds)Advanced Metaheuristic Methods in Big Data Retrieval and Analytics, 1–26.. IGI Global, Hershey, PA.

[7] Al-Joboury IM, Hemiary EH (2018) Internet of Things Architecture Based Cloud for Healthcare. Iraqi J Inf Commun Technol 1(1):18–26.

[8] Catarinucci L, De Donno D, Mainetti L, Palano L, Patrono L, Stefanizzi ML, Tarricone L (2015) An IoT-aware architecture for smart healthcare systems. IEEE Internet Things J 2(6):515–526.

[9] Yu L, Lu Y, Zhu X (2012) Smart hospital based on internet of things. J Netw 7(10):1654.

[10] Mahmoud R, Yousuf T, Aloul F, Zualkernan I (2015) Internet of things (IoT) security: Current status, challenges and prospective measures In: 2015 10th International Conference for Internet Technology and Secured Transactions (ICITST), 336–341.. IEEE, London.

[11] Yuehong Y, Zeng Y, Chen X, Fan Y (2016) The internet of things in healthcare: An overview. J Ind Inf Integr 1:3–13.

[12] Khan R, Khan S, Zaheer R, Khan S (2012) Future internet: The internet of things architecture, possible applications and key challenges In: 2012 10th International Conference on Frontiers of Information Technology, vol. 10, 257–260.. IEEE, Islamabad.

[13] Huxtable J, Schaefer D (2016) On servitization of the manufacturing industry in the UK. Procedia CIRP 52(1):46–51.

[14] Da Xu L, He W, Li S (2014) Internet of things in industries: A survey. IEEE Trans Ind Inform 10(4):2233–2243.

[15] Karagiannis V, Chatzimisios P, Vazquez-Gallego F, Alonso-Zarate J (2015) A survey on application layer protocols for the internet of things. Trans IoT Cloud Comput 3(1):11–17.

[16] Muralidharan S, Roy A, Saxena N (2016) An exhaustive review on Internet of things from Korea’s perspective. Wirel Pers Commun 90(3):1463–1486.

[17] Park A, Chang H, Lee KJ (2017) Action research on development and application of Internet of Things services in hospital. Healthc Inform Res 23(1):25–34. 28261528 10.4258/hir.2017.23.1.25 PMC5334128

[18] Gubbi J, Buyya R, Marusic S, Palaniswami M (2013) Internet of Things (IoT): A vision, architectural elements, and future directions. Futur Gener Comput Syst 29(7):1645–1660.

[19] Crowley ST, Belcher J, Choudhury D, Griffin C, Pichler R, Robey B, Rohatgi R, Mielcarek B (2017) Targeting access to kidney care via telehealth: The VA experience. Adv Chronic Kidney Dis 24(1):22–30. 28224939 10.1053/j.ackd.2016.11.005

[20] Rashed A, Ibrahim A, Adel A, Mourad B, Hatem A, Magdy M, Elgaml N, Khattab A (2017) Japan-Africa Conference on Electronics, Communications, and Computers (JAC-ECC), 160–163.. IEEE, Alexandria.

[21] Qureshi F, Krishnan S (2018) Wearable hardware design for the internet of medical things (IoMT). Sensors 18(11):3812. 30405026 10.3390/s18113812 PMC6263646

[22] Das PK, Zhu F, Chen S, Luo C, Ranjan P, Xiong G (2019) Smart medical healthcare of Internet of medical things (IOMT): application of non-contact sensing In: 2019 14th IEEE Conference on Industrial Electronics and Applications (ICIEA), 375–380.. IEEE, Xi’an.

[23] Pu Q, Gupta S, Gollakota S, Patel S (2013) Whole-home gesture recognition using wireless signals In: Proceedings of the 19th Annual International Conference on Mobile Computing & Networking, 27–38.. Association for Computing Machinery, New York.

[24] Islam MZ, Nahiyan KT, Kiber MA (2015) A motion detection algorithm for video-polysomnography to diagnose sleep disorder In: 2015 18th International Conference on Computer and Information Technology (ICCIT), 272–275.. IEEE, Dhaka.

[25] Pazienza A, Anglani R, Mallardi G, Fasciano C, Noviello P, Tatulli C, Vitulano F (2020) Adaptive critical care intervention in the internet of medical things In: 2020 IEEE Conference on Evolving and Adaptive Intelligent Systems (EAIS), 1–8.. IEEE, Bari.

[26] Ning Z, Dong P, Wang X, Hu X, Guo L, Hu B, Guo Y, Qiu T, Kwok R (2020) Mobile edge computing enabled 5G health monitoring for Internet of medical things: A decentralized game theoretic approach. IEEE J Sel Areas Commun 38:1–16.

[27] Dong P, Ning Z, Obaidat MS, Jiang X, Guo Y, Hu X, Hu B, Sadoun B (2020) Edge computing based healthcare systems: Enabling decentralized health monitoring in Internet of medical Things. IEEE Netw 34:254–261.

[28] Wang EK, Chen CM, Hassan MM, Almogren A (2020) A deep learning based medical image segmentation technique in Internet-of-Medical-Things domain. Futur Gener Comput Syst 108:135–144.

[29] Manimurugan S, Al-Mutairi S, Aborokbah MM, Chilamkurti N, Ganesan S, Patan R (2020) Effective attack detection in internet of medical things smart environment using a deep belief neural network. IEEE Access 8:77396–77404.

[30] Lee S, Shi Q, Lee C (2019) From flexible electronics technology in the era of IoT and artificial intelligence toward future implanted body sensor networks. APL Mater 7(3):031302.

[31] Qadri YA, Nauman A, Zikria YB, Vasilakos AV, Kim SW (2020) The future of healthcare internet of things: a survey of emerging technologies. IEEE Commun Surv Tutorials 22(2):1121–1167.

[32] Mohammed M, Syamsudin H, Al-Zubaidi S, AKS RR, Yusuf E (2020) Novel COVID-19 detection and diagnosis system using IOT based smart helmet. Int J Psychosoc Rehabil 24(7):2296–2303.

[33] Vaishya R, Javaid M, Khan IH, Haleem A (2020) Artificial Intelligence (AI) applications for COVID-19 pandemic. Diabetes Metab Syndr Clin Res Rev 14:337–339. 10.1016/j.dsx.2020.04.012 PMC7195043 32305024

[34] Singh RP, Javaid M, Haleem A, Suman R (2020) Internet of things (IoT) applications to fight against COVID-19 pandemic. Diabetes Metab Syndr Clin Res Rev 14:521–524. 10.1016/j.dsx.2020.04.041 PMC7198990 32388333

[35] Tun SYY, Madanian S, Mirza F (2020) Internet of things (IoT) applications for elderly care: a reflective review. Aging Clin Exp Res 32:1–13. 32277435 10.1007/s40520-020-01545-9

[36] Abou-Nassar EM, Iliyasu AM, El-Kafrawy PM, Song O-Y, Bashir AK, Abd El-Latif AA (2020) DITrust chain: towards blockchain-based trust models for sustainable healthcare IoT systems. IEEE Access 8:111223–111238.

[37] Islam M, Usman M, Mahmood A, Abbasi AA, Song O-Y (2020) Predictive analytics framework for accurate estimation of child mortality rates for Internet of Things enabled smart healthcare systems. Int J Distributed Sens Netw 16(5):1550147720928897.

[38] Zhou X, Liang W, Kevin I, Wang K, Wang H, Yang LT, Jin Q (2020) Deep learning enhanced human activity recognition for internet of healthcare things. IEEE Int Things J 7:6429–6438.

[39] Ilin I, Iliyaschenko O, Konradi A (2018) Business model for smart hospital health organization In: SHS Web of Conferences, vol. 44, 00041.. EDP Sciences, St. Petersburg.

[40] Dhariwal K, Mehta A (2017) Architecture and plan of smart hospital based on Internet of Things (IOT). Int Res J Eng Technol 4(4):1976–1980.

[41] Hassanalieragh M, Page A, Soyata T, Sharma G, Aktas M, Mateos G, Kantarci B, Andreescu S (2015) Health monitoring and management using Internet-of-Things (IoT) sensing with cloud-based processing: Opportunities and challenges In: 2015 IEEE International Conference on Services Computing, 285–292.. IEEE, New York.

[42] Lee J, You K, Jang J (2011) Design and implementation of agent based SPC monitoring system. Int J Ind Eng Comput 18(8):404–413.

[43] Chen M, Ma Y, Song J, Lai C-F, Hu B (2016) Smart clothing: Connecting human with clouds and big data for sustainable health monitoring. Mob Netw Appl 21(5):825–845.

[44] Merilahti J, Viramo P, Korhonen I (2015) Wearable monitoring of physical functioning and disability changes, circadian rhythms and sleep patterns in nursing home residents. IEEE J Biomed Health Inform 20(3):856–864. 25861091 10.1109/JBHI.2015.2420680

[45] Naranjo-Hernández D, Roa LM, Reina-Tosina J, Estudillo-Valderrama MA (2012) SoM: a smart sensor for human activity monitoring and assisted healthy ageing. IEEE Trans Biomed Eng 59(11):3177–3184. 23086195 10.1109/TBME.2012.2206384

[46] Pang Z (2013) Technologies and Architectures of the Internet-of-Things (IoT) for Health and Well-being. PhD thesis, KTH Royal Institute of Technology.

[47] Hossain MS, Muhammad G (2016) Healthcare big data voice pathology assessment framework. IEEE Access 4:7806–7815.

[48] Demirkan H (2013) A smart healthcare systems framework. IT Prof 15(5):38–45.

[49] Liu S, Li W, Liu K (2014) Pragmatic oriented data interoperability for smart healthcare information systems In: 2014 14th IEEE/ACM International Symposium on Cluster, Cloud and Grid Computing, 811–818.. IEEE, Chicago.

[50] Della Vecchia G, Gallo L, Esposito M, Coronato A (2012) An infrastructure for smart hospitals. Multimed Tools Appl 59(1):341–362.

[51] Patsakis C, Venanzio R, Bellavista P, Solanas A, Bouroche M (2014) Personalized medical services using smart cities’ infrastructures In: 2014 IEEE International Symposium on Medical Measurements and Applications (MeMeA), 1–5.. IEEE, Lisbon.

[52] Kim YT, Jeong YS, Park GC (2016) Smart healthcare service model for efficient management of patient at a hospital outpatient visits. Indian J Sci Technol 9(44):105098.

[53] Santos A, Macedo J, Costa A, Nicolau MJ (2014) Internet of things and smart objects for M-health monitoring and control. Procedia Technol 16:1351–1360.

[54] Shirehjini AAN, Yassine A, Shirmohammadi S (2012) Equipment location in hospitals using RFID-based positioning system. IEEE Trans Inf Technol Biomed 16(6):1058–1069. 24218700 10.1109/titb.2012.2204896

[55] Bardram JE (2004) Applications of context-aware computing in hospital work: examples and design principles In: Proceedings of the 2004 ACM Symposium on Applied Computing, 1574–1579.. Association for Computing Machinery, New York.

[56] Ming B, Shuo T, Mingsan M, Jiaojiao J, Weiyun X (2015) Big data applications in traditional Chinese medicine research. Int J Serv Technol Manag 21(4-6):294–300.

[57] Kim HH, Ha KN, Lee S, Lee KC (2009) Resident location-recognition algorithm using a Bayesian classifier in the PIR sensor-based indoor location-aware system. IEEE Trans Syst Man Cybern Part C Appl Rev 39(2):240–245.

[58] Guo J, Chen R, Tsai JJP (2017) A survey of trust computation models for service management in internet of things systems. Comput Commun 97:1–14.

[59] Al-Qaseemi SA, Almulhim HA, Almulhim MF, Chaudhry SR (2016) IoT architecture challenges and issues: Lack of standardization In: 2016 Future Technologies Conference (FTC), 731–738.. IEEE, San Francisco.

[60] Banu NMM, Sujatha C (2017) IoT architecture a comparative study. Int J Pur Appl Math 117(8):45–49.

[61] Osimani F, Stecanella B, Capdehourat G, Etcheverry L, Grampín E (2019) Managing devices of a one-to-one computing educational program using an IoT infrastructure. Sensors 19(1):70. 10.3390/s19010070 PMC6339153 30585214

[62] Kumar NM, Mallick PK (2018) The Internet of Things: Insights into the building blocks, component interactions, and architecture layers. Procedia Comput Sci 132:109–117.

[63] Alhazmi O (2018) A survivable internet of things scheme. J Adv Res Comput Appl 13(1):19–26.

[64] Zyrianoff I, Heideker A, Silva D, Kleinschmidt J, Soininen J-P, Salmon Cinotti T, Kamienski C (2020) Architecting and deploying IoT smart applications: A performance–oriented approach. Sensors 20(1):84. 10.3390/s20010084 PMC6983202 31877812

[65] Khowaja SA, Setiawan F, Prabono AG, Yahya BN, Lee S-L (2016) An effective threshold based measurement technique for fall detection using smart devices. Int J Ind Eng Comput 23(5):332–348.

[66] Qi J, Yang P, Min G, Amft O, Dong F, Xu L (2017) Advanced internet of things for personalised healthcare systems: A survey. Pervasive Mob Comput 41:132–149.

[67] Medronic. https://www.medtronic.com/us-en/healthcare-professionals/products.html.

[68] Shahmiri S (2016) Wearing your data on your sleeve: Wearables, the FTC, and the privacy implications of this new technology. Tex Rev Ent Sports L 18:25.

[69] Farhan L, Kharel R (2019) Internet of things: vision, future directions and opportunities. In: Mukhopadhyay SC, Jayasundera KP, Postolache OA (eds), 331–347.. Springer, Cham.

[70] Lan L, Shi R, Wang B, Zhang L (2019) An iot unified access platform for heterogeneity sensing devices based on edge computing. IEEE Access 7:44199–44211.

[71] Greco L, Ritrovato P, Xhafa F (2019) An edge-stream computing infrastructure for real-time analysis of wearable sensors data. Futur Gener Comput Syst 93:515–528.

[72] Hamidi H (2019) An approach to develop the smart health using Internet of Things and authentication based on biometric technology. Futur Gener Comput Syst 91:434–449.

[73] Xiao B, Rahmani R, Li Y, Kanter T (2017) Edge-based interoperable service-driven information distribution for intelligent pervasive services. Pervasive Mob Comput 40:359–381.

[74] Tolba A, Al-Makhadmeh Z (2020) A recurrent learning method based on received signal strength analysis for improving wireless sensor localization. Circ Syst Sig Process 39(2):1019–1037.

[75] Alarifi A, Tolba A (2019) Optimizing the network energy of cloud assisted internet of things by using the adaptive neural learning approach in wireless sensor networks. Comput Ind 106:133–141.

[76] Zhou YM, Wagner D, Nuckols K, Heimgartner R, Correia C, Clarke M, Orzel D, O’Neill C, Solinsky R, Paganoni S, *et al* (2019) Soft robotic glove with integrated sensing for intuitive grasping assistance post spinal cord injury In: 2019 International Conference on Robotics and Automation (ICRA), 9059–9065.. IEEE, Montreal.

[77] Chung HU, Rwei AY, Hourlier-Fargette A, Xu S, Lee K, Dunne EC, Xie Z, Liu C, Carlini A, Kim DH, *et al* (2020) Skin-interfaced biosensors for advanced wireless physiological monitoring in neonatal and pediatric intensive-care units. Nat Med 26(3):418–429. 32161411 10.1038/s41591-020-0792-9 PMC7315772

[78] Wen F, He T, Liu H, Chen H-Y, Zhang T, Lee C (2020) Advances in chemical sensing technology for enabling the next-generation self-sustainable integrated wearable system in the IoT era. Nano Energy 78:105155.

[79] Robles T, Bordel B, Alcarria R, Sánchez-de-Rivera D (2020) Enabling trustworthy personal data protection in eHealth and well-being services through privacy-by-design. Int J Distrib Sens Netw 16(5):1550147720912110.

[80] Sun J, Xiong H, Liu X, Zhang Y, Nie X, Deng RH (2020) lightweight and privacy-aware fine-grained access control for IoT-oriented smart health. IEEE Int Things J 7:6566–6575.

[81] Schneble CO, Elger BS, Shaw DM (2020) All our data will be health data one day: the need for universal data protection and comprehensive consent. J Med Internet Res 22(5):16879. 10.2196/16879 PMC7290498 32463372

[82] Yang M, Guo J, Zhao Z, Xu T, Bai L (2020) Teenager health oriented data security and privacy protection research for smart wearable device. Procedia Comput Sci 174:333–339.

[83] Tahir H, Tahir R, McDonald-Maier K (2018) On the security of consumer wearable devices in the internet of things. PloS ONE 13(4):0195487. 10.1371/journal.pone.0195487 PMC5905955 29668756

[84] Mao B, Kawamoto Y, Kato N (2020) AI-based joint optimization of QoS and security for 6G energy harvesting internet of things. IEEE Int Things J 8:142875–142891.

[85] Bello O, Zeadally S (2014) Intelligent device-to-device communication in the internet of things. IEEE Syst J 10(3):1172–1182.

[86] Madakam S, Ramaswamy R, Tripathi S (2015) Internet of Things (IoT): A literature review. J Comput Commun 3(05):164.

[87] Kayal P, Perros H (2017) A comparison of IoT application layer protocols through a smart parking implementation In: 2017 20th Conference on Innovations in Clouds, Internet and Networks (ICIN), 331–336.. IEEE, Paris.

[88] Castellani AP, Gheda M, Bui N, Rossi M, Zorzi M (2011) Web Services for the Internet of Things through CoAP and EXI In: 2011 IEEE International Conference on Communications Workshops (ICC), 1–6.. IEEE, Kyoto.

[89] Thangavel D, Ma X, Valera A, Tan H-X, Tan CK-Y (2014) Performance evaluation of MQTT and CoAP via a common middleware In: 2014 IEEE Ninth International Conference on Intelligent Sensors, Sensor Networks and Information Processing (ISSNIP), 1–6.. IEEE, Singapore.

[90] Kirsche M, Klauck R (2012) Unify to bridge gaps: Bringing XMPP into the Internet of Things In: 2012 IEEE International Conference on Pervasive Computing and Communications Workshops, 455–458.. IEEE, Lugano.

[91] Fielding RT, Taylor RN (2000) Architectural styles and the design of network-based software architectures. vol. 7. University of California, Irvine.

[92] Vinoski S (2006) Advanced message queuing protocol. IEEE Internet Comput 10(6):87–89.

[93] Basem ALM, Ali H (2017) Data Distribution Service (DDS) based implementation of Smart grid devices using ANSI C12. 19 standard. Procedia Comput Sci 110:394–401.

[94] Bansal M, *et al* (2020) Application layer protocols for internet of healthcare things (IoHT) In: 2020 Fourth International Conference on Inventive Systems and Control (ICISC), 369–376.. IEEE, Islamic Republic of Iran.

[95] Naik N (2017) Choice of effective messaging protocols for IoT systems: MQTT, CoAP, AMQP and HTTP In: 2017 IEEE International Systems Engineering Symposium (ISSE), 1–7.. IEEE, Vienna.

[96] Gluhak A, Krco S, Nati M, Pfisterer D, Mitton N, Razafindralambo T (2011) A survey on facilities for experimental internet of things research. IEEE Commun Mag 49(11):58–67.

[97] Pothuganti K, Chitneni A (2014) A comparative study of wireless protocols: Bluetooth, UWB, ZigBee, and Wi-Fi. Adv Electron Electr Eng 4(6):655–662.

[98] Deshmukh N, Matte PN (2014) An application of ZigBee for machine health monitoring. Int J Innov Res Sci Eng Technol 3(3):10437–10444.

[99] Khan M, Din S, Jabbar S, Gohar M, Ghayvat H, Mukhopadhyay S (2016) Context-aware low power intelligent SmartHome based on the Internet of things. Comput Electr Eng 52:208–222.

[100] Kumar A, Amarlingam M, Rajalakshmi P (2017) Random node sampling approach for energy efficient data gathering in wireless sensor networks In: 2017 IEEE Region 10 Symposium (TENSYMP), 1–5.. IEEE, Cochin.

[101] Salwe SS, Naik KK (2019) Heterogeneous wireless network for IoT applications. IETE Tech Rev 36(1):61–68.

[102] Froiz-Míguez I, Fernández-Caramés TM, Fraga-Lamas P, Castedo L (2018) Design, implementation and practical evaluation of an IoT home automation system for fog computing applications based on MQTT and ZigBee-WiFi sensor nodes. Sensors 18(8):2660. 30104529 10.3390/s18082660 PMC6111259

[103] Karimi K, Krit S (2019) Internet of thing for smart home system using web services and android application In: Smart Network Inspired Paradigm and Approaches in IoT Applications, 191–202.. Springer, Singapore.

[104] Sharma SK, Bogale TE, Chatzinotas S, Wang X, Le LB (2016) Physical layer aspects of wireless IoT In: 2016 International Symposium on Wireless Communication Systems (ISWCS), 304–308.. IEEE, Poznan.

[105] Lu X, Wang P, Niyato D, Kim DI, Han Z (2015) Wireless charging technologies: Fundamentals, standards, and network applications. IEEE Commun Surv Tutorials 18(2):1413–1452.

[106] Chakkor S, Cheikh EA, Baghouri M, Hajraoui A (2014) Comparative performance analysis of wireless communication protocols for intelligent sensors and their applications. arXiv preprint arXiv:1409.6884 5:76–85.

[107] Chaabouni N, Mosbah M, Zemmari A, Sauvignac C, Faruki P (2019) Network intrusion detection for IoT security based on learning techniques. IEEE Commun Surv Tutorials 21(3):2671–2701.

[108] Rizvi S, Orr RJ, Cox A, Ashokkumar P, Rizvi MR (2020) Identifying the attack surface for IoT network. Internet of Things 9:100162.

[109] Alshehri MD, Hussain FK (2019) A fuzzy security protocol for trust management in the internet of things (Fuzzy-IoT). Computing 101(7):791–818.

[110] Fysarakis K, Askoxylakis I, Soultatos O, Papaefstathiou I, Manifavas C, Katos V (2016) Which IoT protocol? Comparing standardized approaches over a common M2M application In: 2016 IEEE Global Communications Conference (GLOBECOM), 1–7.. IEEE, Washington.

[111] Papcun P, Kajati E, Cupkova D, Mocnej J, Miskuf M, Zolotova I (2020) Edge-enabled IoT gateway criteria selection and evaluation 32:5219.

[112] Al-Turjman F (2017) Price-based data delivery framework for dynamic and pervasive IoT. Pervasive Mob Comput 42:299–316.

[113] Mohanraj R (2020) Optimized load centroid and Rabin onion secured routing in wireless sensor network for IoT. Glob J Comput Sci Technol 20:1–13.

[114] Yang J, Han Y, Wang Y, Jiang B, Lv Z, Song H (2020) Optimization of real-time traffic network assignment based on IoT data using DBN and clustering model in smart city. Futur Gener Comput Syst 108:976–986.

[115] He Y, Zhang S, Tang L, Ren Y (2020) Large scale resource allocation for the internet of things network based on ADMM. IEEE Access 8:57192–57203.

[116] Fang H, Qi A, Wang X (2020) Fast authentication and progressive authorization in large-scale IoT: How to leverage ai for security enhancement. IEEE Network 34(3):24–29.

[117] Iwendi C, Maddikunta PKR, Gadekallu TR, Lakshmanna K, Bashir AK, Piran MJ (2020) A metaheuristic optimization approach for energy efficiency in the IoT networks. Softw Pract Experience:1–14.

[118] Dhanvijay MM, Patil SC (2019) Internet of Things: A survey of enabling technologies in healthcare and its applications. Comput Netw 153:113–131.

[119] Hong HJ (2017) From cloud computing to fog computing: unleash the power of edge and end devices In: 2017 IEEE International Conference on Cloud Computing Technology and Science (CloudCom), 331–334.. IEEE, Hong Kong.

[120] Knorr E, Gruman G (2008) What cloud computing really means. InfoWorld 7:20–20.

[121] Liu C, Gao J, Li Y, Wang H, Chen Z (2020) Studying gas exceptions in blockchain-based cloud applications. J Cloud Comput 9(1):1–25.

[122] Shih YY, Chung WH, Pang AC, Chiu TC, Wei HY (2016) Enabling low-latency applications in fog-radio access networks. IEEE Network 31(1):52–58.

[123] Guan Z, Zhang Y, Zhu L, Wu L, Yu S (2019) EFFECT: An efficient flexible privacy-preserving data aggregation scheme with authentication in smart grid. Sci China Inf Sci 62(3):32103.

[124] Kazarian A, Teslyuk V, Tykhan M, Mashevska M (2019) Usage Of SaaS software delivery model in intelligent house system. Przeglad Elektrotechniczny 95(7):38–41.

[125] Ribas M, Lima AS, de Souza JN, de Carvalho Sousa FR, Moreira LO (2016) A platform as a service billing model for cloud computing management approaches. IEEE Lat Am Trans 14(1):267–280.

[126] Xu S, Xin Y, Zhu H, Luo S, Chen Y (2019) A authentication and access authorization mechanism on the PaaS platform In: 2019 IEEE Symposium Series on Computational Intelligence (SSCI), 893–900.. IEEE, Xiamen.

[127] Ahmed A, Zakariae T, *et al* (2018) IaaS cloud model security issues on behalf cloud provider and user security behaviors. Procedia Comput Sci 134:328–333.

[128] Mell P, Grance T (2011) The NIST definition of cloud computing.

[129] Stojmenovic I, Wen S (2014) The fog computing paradigm: Scenarios and security issues In: 2014 Federated Conference on Computer Science and Information Systems, 1–8.. IEEE, Warsaw.

[130] Tönjes R, Barnaghi P, Ali M, Mileo A, Hauswirth M, Ganz F, Ganea S, Kjærgaard B, Kuemper D, Nechifor S, *et al* (2014) Real time IoT stream processing and large-scale data analytics for smart city applications In: poster Session, European Conference on Networks and Communications.. sn, Porto.

[131] Yousefpour A, Fung C, Nguyen T, Kadiyala K, Jalali F, Niakanlahiji A, Kong J, Jue JP (2019) All one needs to know about fog computing and related edge computing paradigms: A complete survey. J Syst Archit 98:289–330.

[132] Shi W, Cao J, Zhang Q, Li Y, Xu L (2016) Edge computing: Vision and challenges. IEEE Internet Things J 3(5):637–646.

[133] Preden JS, Tammemäe K, Jantsch A, Leier M, Riid A, Calis E (2015) The benefits of self-awareness and attention in fog and mist computing. Computer 48(7):37–45.

[134] Uehara M (2017) Mist computing: linking cloudlet to fogs In: International Conference on Computational Science/Intelligence & Applied Informatics, 201–213.. Springer, Macau.

[135] Wu M, Lu TJ, Ling FY, Sun J, Du HY (2010) Research on the architecture of Internet of Things In: 2010 3rd International Conference on Advanced Computer Theory and Engineering (ICACTE), vol 5, 5–484.. IEEE, Chengdu.

[136] Liao Y, Qi H, Li W (2012) Load-balanced clustering algorithm with distributed self-organization for wireless sensor networks. IEEE Sensors J 13(5):1498–1506.

[137] Urbano O, Perles A, Pedraza C, Rubio-Arraez S, Castelló ML, Ortola MD, Mercado R (2020) Cost-effective implementation of a temperature traceability system based on smart RFID Tags and IoT services. Sensors 20(4):1163. 32093218 10.3390/s20041163 PMC7071464

[138] Leban A, Košir P (2019) Aspects of Implementing GIS as a Centralised System in an Enterprise IT/OT Environment In: 25th International Conference on Electricity Distribution, Madrid.

[139] Isikdag U (2015) BIM and IoT: a synopsis from GIS perspective. Int Arch Photogramm Remote Sens Spat Inf Sci 40:33.

[140] Chinnaswamy A, Papa A, Dezi L, Mattiacci A (2019) Big data visualisation, geographic information systems and decision making in healthcare management. Manag Decis 57:1937–1959.

[141] Chung Y, Bagheri N, Salinas-Perez JA, Smurthwaite K, Walsh E, Furst M, Rosenberg S, Salvador-Carulla L (2020) Role of visual analytics in supporting mental healthcare systems research and policy: A systematic scoping review. Int J Inf Manag 50:17–27.

[142] Tuli S, Basumatary N, Gill SS, Kahani M, Arya RC, Wander GS, Buyya R (2020) Healthfog: An ensemble deep learning based smart healthcare system for automatic diagnosis of heart diseases in integrated iot and fog computing environments. Futur Gener Comput Syst 104:187–200.

[143] Patan R, Ghantasala GSP, Sekaran R, Gupta D, Ramachandran M (2020) Smart healthcare and quality of service in IoT using grey filter convolutional based cipher physical system. Sustain Cities Soc 59:102141.

[144] Cheng JCP, Chen W, Chen K, Wang Q (2020) Data-driven predictive maintenance planning framework for MEP components based on BIM and IoT using machine learning algorithms. Autom Constr 112:103087.

[145] Jaigirdar FT, Rudolph C, Bain C (2019) Can I trust the data I see? A physician’s concern on medical data in IoT health architectures In: Proceedings of the Australasian Computer Science Week Multiconference, 1–10.. Association for Computing Machinery, New York.

[146] Hu R, Yan Z, Ding W, Yang LT (2020) A survey on data provenance in IoT. World Wide Web 23(2):1441–1463.

[147] Elkhodr M, Alsinglawi B (2020) Data provenance and trust establishment in the Internet of Things. Secur Priv 3(3):99.

[148] Olokodana IL, Mohanty SP, Kougianos E, Olokodana OO (2020) Real-time automatic seizure detection using ordinary Kriging method in an edge-IoMT computing paradigm. SN Comput Sci 1(5):1–15.

[149] Yang J, Ma C, Jiang B, Ding G, Zheng G, Wang H (2020) Joint optimization in cached-enabled heterogeneous network for efficient industrial IoT. IEEE J Sel Areas Commun 38(5):831–844.

[150] Shafique K, Khawaja BA, Sabir F, Qazi S, Mustaqim M (2020) Internet of things (IoT) for next-generation smart systems: A review of current challenges, future trends and prospects for emerging 5G-IoT scenarios. IEEE Access 8:23022–23040.

[151] Zyane A, Bahiri MN, Ghammaz A (2020) IoTScal-H: Hybrid monitoring solution based on cloud computing for autonomic middleware-level scalability management within IoT systems and different SLA traffic requirements. Int J Commun Syst 33(14):4495.

[152] Aftab N, Zaidi SAR, McLernon D (2020) Scalability analysis of multiple LoRa gateways using stochastic geometry. Internet of Things 9:100132.

[153] Preet IK, Saini KS (2020) A Systematic Evaluation of Literature on Internet of Things (IoT) and Smart Technologies with Multiple Dimensions. J Technol Manag Growing Econ 11(1):1–10.

[154] Rafique W, Qi L, Yaqoob I, Imran M, ur Rasool R, Dou W (2020) Complementing IoT Services through Software Defined Networking and Edge Computing: A Comprehensive Survey. IEEE Commun Surv Tutorials 22:1761–1804.

[155] Zang W, Miao F, Gravina R, Sun F, Fortino G, Li Y (2020) CMDP-based intelligent transmission for wireless body area network in remote health monitoring. Neural Comput & Applic 32(3):829–837.

[156] Yang S, Ma L, Jia S, Qin D (2020) A Novel Markov model-based low-power and secure multihop routing mechanism. J Sensors 2019:1–11.

[157] Emam A, Abdellatif AA, Mohamed A, Harras KA (2019) EdgeHealth: An energy-efficient edge-based remote mHealth monitoring system In: 2019 IEEE Wireless Communications and Networking Conference (WCNC), 1–7.. IEEE, Marrakech.

[158] He Z, Zhang Y, Tak B, Peng L (2019) Green fog planning for optimal internet-of-thing task scheduling. IEEE Access 8:1224–1234.

[159] Aburukba RO, AliKarrar M, Landolsi T, El-Fakih K (2020) Scheduling Internet of Things requests to minimize latency in hybrid Fog–Cloud computing. Futur Gener Comput Syst 111:539–551.

[160] Jaleel A, Mahmood T, Hassan MA, Bano G, Khurshid SK (2020) Towards medical data interoperability through collaboration of healthcare devices. IEEE Access 8:132302–132319.

[161] Fouad H, Mahmoud NM, El Issawi MS, Al-Feel H (2020) Distributed and scalable computing framework for improving request processing of wearable IoT assisted medical sensors on pervasive computing system. Comput Commun 151:257–265.

[162] Tsang L, Kracov DA, Mulryne J, Strom L, Perkins N, Dickinson R, Wallace VM, Jones B (2017) The impact of artificial intelligence on medical innovation in the European Union and United States. Intellect Prop Technol Law J 29:3–12.

[163] Keleş A, Keleş A, Yavuz U (2011) Expert system based on neuro-fuzzy rules for diagnosis breast cancer. Expert Syst Appl 38(5):5719–5726.

[164] Paydar S, Pourahmad S, Azad M, Bolandparvaz S, Taheri R, Ghahramani Z, Zamani A, Jeddi M, Karimi F, Dabbaghmanesh MH, Shams M, Abbasi HR (2016) The evolution of a malignancy risk prediction model for thyroid nodules using the artificial neural network. Middle East J Cancer 7(1):47–52.

[165] Krupitzer C, Roth FM, VanSyckel S, Schiele G, Becker C (2015) A survey on engineering approaches for self-adaptive systems. Pervasive Mob Comput 17:184–206.

[166] Minerva R, Lee GM, Crespi N (2020) Digital twin in the IoT context: a survey on technical features, scenarios, and architectural models. Proc IEEE 108(10):1785–824.

[167] Li G, Dong M, Yang LT, Ota K, Wu J, Li J (2020) Preserving edge knowledge sharing among IoT services: A blockchain-based approach. IEEE Trans Emerg Top Comput Intell 4(5):653–665.

[168] Xhafa F, Kilic B, Krause P (2020) Evaluation of IoT stream processing at edge computing layer for semantic data enrichment. Futur Gener Comput Syst 105:730–736.

[169] Rathee G, Garg S, Kaddoum G, Choi BJ (2020) A decision-making model for securing IoT devices in smart industries. IEEE Trans Ind Inform:1–9.

[170] Alonso RS, Sittón-Candanedo I, García Ó, Prieto J, Rodríguez-González S (2020) An intelligent Edge-IoT platform for monitoring livestock and crops in a dairy farming scenario. Ad Hoc Netw 98:102047.

[171] Guo X, Lin H, Wu Y, Peng M (2020) A new data clustering strategy for enhancing mutual privacy in healthcare IoT systems. Futur Gener Comput Syst 113:407–417.

[172] Yacchirema D, de Puga JS, Palau C, Esteve M (2019) Fall detection system for elderly people using IoT and ensemble machine learning algorithm. Pers Ubiquit Comput 23(5-6):801–817.

[173] Kaur P, Kumar R, Kumar M (2019) A healthcare monitoring system using random forest and internet of things (IoT). Multimed Tools Appl 78(14):19905–19916.

[174] Dash S, Acharya BR, Mittal M, Abraham A, Kelemen A (2020) Deep learning techniques for biomedical and health informatics. Springer, Cham.

[175] Sanin C, Haoxi Z, Shafiq I, Waris MM, de Oliveira CS, Szczerbicki E (2019) Experience based knowledge representation for internet of things and cyber physical systems with case studies. Futur Gener Comput Syst 92:604–616.

[176] Yan H, Huo H, Xu Y, Gidlund M (2010) Wireless sensor network based E-health system-implementation and experimental results. IEEE Trans Consum Electron 56(4):2288–2295.

[177] Matusitz J, Breen GM (2007) Telemedicine: Its effects on health communication. Health Commun 21(1):73–83. 17461754 10.1080/10410230701283439

[178] Price ND, Edelman LB, Lee I, Yoo H, Hwang D, Carlson G, Galas DJ, Heath JR, Hood L (2009) Systems biology and systems medicine. In: Ginsburg G Willard H (eds)In ‘Genomic and Personalized Medicine: From Principles to Practice’, 74–85.. Elsevier, Amsterdam.

[179] Hood L, Flores M (2012) A personal view on systems medicine and the emergence of proactive P4 medicine: predictive, preventive, personalized and participatory. New Biotechnol 29(6):613–624. 10.1016/j.nbt.2012.03.004 22450380

[180] Hood L, Heath JR, Phelps ME, Lin B (2004) Systems biology and new technologies enable predictive and preventative medicine. Science 306(5696):640–643. 15499008 10.1126/science.1104635

[181] Stocker C, Marzi LM, Matula C, Schantl J, Prohaska G, Brabenetz A, Holzinger A (2014) Enhancing patient safety through human-computer information retrieval on the example of german-speaking surgical reports In: 2014 25th International Workshop on Database and Expert Systems Applications, 216–220.. IEEE, Munich.

[182] Holzinger A, Dehmer M, Jurisica I (2014) Knowledge discovery and interactive data mining in bioinformatics-state-of-the-art, future challenges and research directions. BMC Bioinformatics 15(6):1–9. 25078282 10.1186/1471-2105-15-S6-I1 PMC4140208

[183] Reddy RV, Murali D, Rajeshwar J (2019) Context-aware middleware architecture for IoT-based smart healthcare applications In: Innovations in Computer Science and Engineering, 557–567.. Springer, Singapore.

[184] Ahmadi H, Arji G, Shahmoradi L, Safdari R, Nilashi M, Alizadeh M (2019) The application of internet of things in healthcare: a systematic literature review and classification. Univ Access Inf Soc 18:1–33.

[185] Carvalho TDPM, de Paiva JC, de MedeirosValentim RA, Silva CBP, de Lima DF, Silva EC (2020) Sabiá: an authentication, authorization, and user data delivery architecture based on user consent for health information systems in Brazil. Res Biomed Eng 36:1–6.

[186] Garg R, *et al* (2019) Existing enabling technologies and solutions for energy management in IoT In: Energy Conservation for IoT Devices, 19–47.. Springer, Singapore.

[187] Berry MJA, Linoff GS (2004) Data mining techniques: for marketing, sales, and customer relationship management. John Wiley & Sons, Indiana.

[188] Neelamegam S, Ramaraj E (2013) Classification algorithm in data mining: An overview. Int J P2P Netw Trends Technol (IJPTT) 4(8):369–374.

[189] Tsiptsis KK, Chorianopoulos A (2011) Data mining techniques in crm: inside customer segmentation. John Wiley & Sons, West Sussex, United Kingdom.

[190] Rajagopal D, *et al* (2011) Customer data clustering using data mining technique. arXiv preprint arXiv:1112.266 3:1–11.

[191] Brossette SE, Sprague AP, Hardin JM, Waites KB, Jones WT, Moser SA (1998) Association rules and data mining in hospital infection control and public health surveillance. J Am Med Inform Assoc 5(4):373–381. 9670134 10.1136/jamia.1998.0050373 PMC61314

[192] Tsai KL, Huang YL, Leu FY, You I, Huang YL, Tsai CH (2018) AES-128 based secure low power communication for LoRaWAN IoT environments. IEEE Access 6:45325–45334.

[193] Gutierrez PJA, Wigard J, Andersen PN, Damgaard HC, Mogensen P (2000) Performance of link adaptation in GPRS networks In: Vehicular Technology Conference Fall 2000. IEEE VTS Fall VTC2000. 52nd Vehicular Technology Conference (Cat. No. 00CH37152), vol 2, 492–499.. IEEE, Montreal.

[194] Song Y, Beznosov K, Leung VCM (2006) Multiple-channel security architecture and its implementation over SSL. EURASIP J Wirel Commun Netw 2006(1):085495.

[195] Farashahi RR, Rashidi B, Sayedi SM (2014) FPGA based fast and high-throughput 2-slow retiming 128-bit AES encryption algorithm. Microelectron J 45(8):1014–1025.

[196] Vermesan O, Bröring A, Tragos E, Serrano M, Bacciu D, Chessa S, Gallicchio C, Micheli A, Dragone M, Saffiotti A, *et al* (2017) Internet of robotic things: converging sensing/actuating, hypoconnectivity, artificial intelligence and IoT Platforms. Cognitive Hyperconnected digital transformation: internet of things intelligence evolution(Vermesan O, Bacquet J, eds.). River Publishers.

[197] Zhu X, Ghahramani Z, Lafferty JD (2003) Semi-supervised learning using gaussian fields and harmonic functions In: Proceedings of the 20th International Conference on Machine Learning (ICML-03), 912–919.. The AAAI Press, Menlo Park.

[198] Zhou J, Li X, Mitri HS (2016) Classification of rockburst in underground projects: comparison of ten supervised learning methods. J Comput Civ Eng 30(5):04016003.

[199] Reddy YCAP, Viswanath P, Reddy BE (2018) Semi-supervised learning: A brief review. Int J Eng Technol 7(1.8):81.

[200] Zha ZJ, Mei T, Wang J, Wang Z, Hua XS (2009) Graph-based semi-supervised learning with multiple labels. J Vis Commun Image Represent 20(2):97–103.

[201] Kibria MG, Chong I (2016) Knowledge-based open Internet of Things service provisioning architecture on beacon-enabled Web of Objects. Int J Distrib Sens Netw 12(9):1550147716660896.

[202] Çalış B (2015) Cooperation & coordination of distributed intelligent agents for manufacturing systems.

[203] Kolozali S, Bermudez-Edo M, Puschmann D, Ganz F, Barnaghi P (2014) A knowledge-based approach for real-time iot data stream annotation and processing In: 2014 IEEE International Conference on Internet of Things (iThings), and IEEE Green Computing and Communications (GreenCom) and IEEE Cyber, Physical and Social Computing (CPSCom), 215–222.. IEEE, Washington.

[204] Banerjee S, Chandra MG (2019) A software framework for procedural knowledge based collaborative data analytics for IoT In: 2019 IEEE/ACM 1st International Workshop on Software Engineering Research & Practices for the Internet of Things (SERP4IoT), 41–48.. IEEE, Montreal.

[205] Amato F, Cozzolino G, Maisto A, Mazzeo A, Moscato V, Pelosi S, Picariello A, Romano S, Sansone C (2015) ABC: a knowledge based collaborative framework for e-health In: 2015 IEEE 1st International Forum on Research and Technologies for Society and Industry Leveraging a Better Tomorrow (RTSI), 258–263.. IEEE, Torino.

